# Schiff Base Heterometallic Complexes and Their Potential Applications

**DOI:** 10.1021/acs.cgd.5c01024

**Published:** 2026-02-09

**Authors:** Jocelyn Pradegan, Aurélien Crochet, Katharina M. Fromm

**Affiliations:** Department of Chemistry, 27211 University of Fribourg, Chemin du Musée 9, Fribourg 1700, Switzerland

## Abstract

A Schiff base ligand, H_2_L, with two distinct compartments to accommodate a transition and an alkaline earth metal ion was used to synthesize two monometallic, [CuL­(H_2_O)­(CH_3_OH)] (**1**) and [NiL­(H_2_O)] (**2**), and six heterometallic complexes, [CuCaL­(NO_3_)_2_] (**3**), [CuSrL­(NO_3_)_2_] (**4**), [CuBaL­(NO_3_)_2_]­(H_2_O) (**5**), [NiCaL­(NO_3_)_2_] (**6**), [NiSrL­(NO_3_)_2_] (**7**), and [NiBaL­(NO_3_)_2_] (**8**), which were characterized by single crystal X-ray diffraction. Their potential as single-source precursors (SSPs) and their antibacterial properties were investigated. After annealing at 1000 °C in air for 1 h, the complexes (**3**), (**4**), (**7**), and (**8**) formed the following mixed metal oxides (MMOs): Ca_2_CuO_3_ for (**3**), CuSrO_2_ and CuSr_2_O_3_ for (**4**), Ni_6.64_Sr_9_O_21_ and Ni_2_Sr_4_O_9_ for (**7**), and Ni_5_Ba_6_O_15_ for (**8**). The mixture of two heterobimetallic complexes (**3**) and (**4**), (**3**) and (**7**), and (**4**) and (**6**) allowed the formation of MMOs containing three different metal ions, such as CuCa_1.50_Sr_0.50_O_3_, CuCa_0.38_Sr_0.62_O_2_, and CaCuSrO_3_. The scanning electron microscopy (SEM) study revealed a sponge-like structure for all the obtained MMOs. The six heterobimetallic complexes showed good solubility in MeOH, DMF, and DMSO. With regard to the antibacterial properties, the protonated ligand and monometallic complex of copper­(II) showed a zone of inhibition (ZOI) that was twice the size of the tested heterobimetallic complexes.

## Graphical abstract

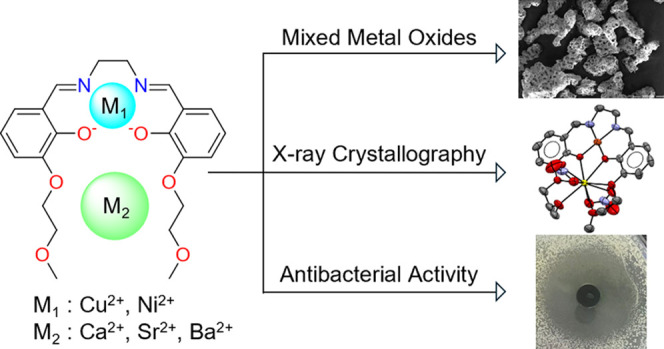

## Introduction

Mixed metal oxides (MMOs) are chemical compounds composed of oxygen atoms and at least two metal cations.^1^ A well-known natural MMO is the perovskite mineral CaTiO_3_, whose name is now used for all compounds with similar crystal structure and general chemical formula ABX_3_, famous for their use in perovskite solar cells.^2^ MMOs have furthermore a wide range of applications in catalysis,^1,3–6^ like the perovskite oxide ABO_3_,^7,8^ semiconductivity,^9–11^ like the In_2_O_3_/SnO_2_ ^12^ semiconductor or pyrochlore oxides,^13^ superconductivity,^14–17^ like the high-temperature superconductors YBa_2_Cu_3_O_7_,^18^ and BiSrCaCu_2_O_*x*_,^19^ or sensing.^20–22^ like the ZnO–SnO_2_ composites.^23,24^ Different synthetic approaches for the formation of MMOs are possible, such as solid-state synthesis,^25^ for which typically grinding, high temperature, and long reaction times are necessary. There are also the sol–gel technique,^26^ and the coprecipitation method,^27^ both using softer conditions.^28^ Another method is based on single source precursors (SSPs), in which several different metal ions are coordinated within one single ligand, allowing for a better control of the stoichiometry.^29,30^

A number of heterometallic complexes were developed as SSPs,^31,32^ many of which are based on Schiff base ligands as the organic part.^33–35^ Schiff Base compounds, particularly with salen-type ligands, are good candidates for the formation of complexes due to their straightforward synthesis.^36^ Indeed, salen-type ligands possess a tetradentate N_2_O_2_ chelate site, which can specifically host a variety of transition metal ions forming stable complexes.^37^ Thanks to their versatility, salen-type ligands can be easily functionalized with various groups containing donor atoms, such as N, O or S, enabling the coordination of additional metal ions.^38^

Schiff base ligands have been widely studied to generate mono- ^39–43^ and multimetallic^44–48^ complexes. The compartmentalization of, e.g., alkaline earth and transition metal ions, which are often found in oxide materials, has been less studied^49–52^ and led to different properties such as luminescence,^53–57^ magnetism,^58–60^ or antimicrobial activities.^61,62^ However, studies of SSPs based on compartmentalized Schiff base ligands with those metal ions are still limited. SSPs based on the association of transition metal ions, such as copper­(I) or (II), and alkaline earth metal ions, like calcium, strontium and barium, within an organic ligand for the formation of MMOs, such as yttrium barium copper oxide (YBCO) or bismuth strontium calcium copper oxide (BSCCO), were reported.^63,64^ Other investigations were based on the replacement of copper by nickel ions in the composition of MMOs.^65,66^

In our research group, previous studies have been conducted on the formation of heterobimetallic complexes based on a salen-type ligand (formed via a condensation reaction of o-vanillin and ethylenediamine). The N_2_O_2_ site was occupied by one transition metal ion (Cu^2+^ or Ni^2+^) and the O_2_O_2_ cavity by either one alkali (Li^+^, Na^+^, K^+^, Rb^+^, and Cs^+^)^21^ or one alkaline earth (Mg^2+^, Ca^2+^, Sr^2+^, and Ba^2+^)^34^ metal ion. Furthermore, always with the same ligand, our research group reported bimetallic complexes, where the N_2_O_2_ cavity is occupied by Cu^2+^ and the O_2_O_2_ cavity is filled with different transition (Ag^+^, Mn^2+^, Cu^2+^, Zn^2+^) or post-transition (Bi^3+^) metal ions.^33^ These bimetallic complexes showed intriguing properties like good antimicrobial action or the formation of nanoscale MMOs.^33^

In this study, we continue the investigation on (hetero)­bimetallic complexes with another derivative of a salen-type ligand, functionalized with ethylene glycol monomethyl ether chains. The obtained mono- and heterobimetallic complexes were characterized by single crystal X-ray diffraction. The heterobimetallic complexes were tested as MMO precursors. Furthermore, as the selected alkaline earth metal ions are biocompatible, the antibacterial properties of the copper­(II)-based complexes were investigated on*Staphylococcus aureus*by a Kirby–Bauer disk diffusion test. By annealing the heterobimetallic complexes or mixture of heterobimetallic complexes at 1000 °C in air for 1 h, heterobi- or trimetallic oxides could be obtained.

## Experimental Section

### Material and Methods

All chemicals were commercially available and used without further purification. All of the experiments were done in air and at room temperature (RT). The ^1^H and ^13^C nuclear magnetic resonance (NMR) spectra were recorded on a Bruker, 400 MHz spectrometer. The electrospray ionization mass spectrometry (ESI-MS) spectra were recorded under positive ion mode on a Bruker, esquire HCT, Ion-Trap Mass Spectrometer. A Mettler Toledo TGA/DSC 3^+^ STAR^e^ System was used for thermogravimetric analyses (TGA). SEM samples were prepared by deposition of the sample powder directly on a carbon-based tape and analyzed without coating. Imaging was done using a Philips XL 30 SFEG working at 20 kV and acquired with the secondary electron detector. Powder X-ray patterns were collected on a STOE, STADI P using Cu–Kα1 radiation (1.540598 Å) or a Bruker, D8 advance using Cu–Kα1 (1.541874 Å). The crystal structure determination of the single crystals was performed on a STOE STADIVARI diffractometer with Cu Kα1 radiation (λ = 1.54186 Å) for complexes **1**, **2**, **3**, **4**, **7** and **8**, and with Ag Kα radiation (λ = 0.56083) for complexes **5** and **6**. A suitable crystal was selected and mounted on a loop with an inert oil (Paratone). The crystal was kept at 250(2) K during data collection. Using Olex2 ^68^ the structure was solved with the SHELXT^69^ structure solution program using Intrinsic Phasing and refined with the SHELXL^70^ refinement package using Least Square minimization. CIF files are available in the Cambridge Crystallographic Data Centre, CCDC-2463279 (**1**), CCDC-2463280 (**2**), CCDC-2463281 (**3**), CCDC-2463282 (**4**), CCDC-2463283 (**5**), CCDC-2463284 (**6**), CCDC-2463285 (**7**), and CCDC-2463286 (**8**).

### Synthesis of 2-Methoxyethyl 4-Methylbenzenesulfonate, L′ (Scheme 1)

The compound was synthesized as described in the literature.^71^ 2-Methoxyethanol (5.0 g, 1.0 eq., 65.7 mmol) was diluted in tetrahydrofuran (THF) (100 mL) in an ice bath. *p*-Toluenesulfonyl chloride (18.8 g, 1.5 equiv, 98.6 mmol) was added when the solution of 2-Methoxyethanol reached 0 °C. The solution was stirred for 5 min at 0 °C. A solution of potassium hydroxide (KOH) 7 M (29.5 g, 8.0 equiv, 525.6 mmol) in distilled water (75 mL) was prepared in an ice bath, since it is an exothermic solution (the high molarity of this solution renders it highly corrosive and should therefore be handled with care and the corresponding personal protective equipment). The KOH solution was added dropwise to the first solution at 0 °C. The solution was stirred at RT overnight. THF was removed under reduced pressure using a rotatory evaporator with a 40 °C bath temperature. A 5 M solution of ammonium chloride (26.75 g) in water (100 mL) was prepared and poured into the solution. The aqueous phase was extracted with dichloromethane (DCM) (5 × 50 mL) using a separatory funnel. The organic phases were collected in a beaker, and subsequently dried magnesium sulfate (ca. 6 g) was added to dry the organic layer. The solution was filtered and evaporated under reduced pressure using a rotatory evaporator with a 40 °C bath temperature. 2-Methoxyethyl 4-methylbenzenesulfonate, L′, was obtained as a transparent pale green oil. Yield: 96%. ^1^H NMR (400 MHz, DMSO-*d*_6_) δ: 7.79 (d, *J* = 7.2 Hz, 2H), 7.48 (d, *J* = 7.4 Hz, 2H), 4.12 (m, 2H), 3.49 (m, 2H), 3.18 (s, 3H), 2.42 (s, 3H). ^13^C NMR (101 MHz, DMSO-*d*_6_) δ: 144.88, 132.40, 130.10, 127.57, 69.69, 69.23, 57.90, 21.03. ESI-MS calculated *m*/*z* for C_10_H_14_O_4_S + Na^+^ ([M + Na]^+^) = 253.1, found 253.2.

### Synthesis of 2-Hydroxy-3-(2-methoxyethoxy)­benzaldehyde, L″ (Scheme 1)

The Williamson ether synthesis reaction was carried out following the literature procedure.^71^ Sodium hydride (NaH) (3.1 g, 2.1 equiv, 76.0 mmol) was poured in a two-neck round-bottom flask with 1 dropping funnel before being purged under argon. Dry dimethyl sulfoxide (DMSO) (10 mL) was added in the two-neck round-bottom flask and placed in an ice bath. 2,3-Dihydroxybenzaldheyde (5.0 g, 1.0 equiv, 36.2 mmol) was deposited in a round-bottom flask with a septum before being purged under argon. Dry DMSO (10 mL) was added in the round-bottom flask. The solution of 2,3-dihydroxybenzaldehyde was taken with a syringe, poured in the dropping funnel, and added dropwise (during 30 min) to the solution of NaH under a flow of argon. After complete addition, the dark orange solution was stirred at room temperature for 90 min, and L′ (8.3 g, 1.0 equiv, 36.0 mmol) was added in one portion. The solution was stirred at room temperature for 150 min. Water (50 mL) was slowly poured to quench the reaction, and the solution was extracted with DCM (3 × 30 mL). The aqueous phase was acidified to pH 2 with 1 M hydrochloric acid (HCl) (15 mL). The aqueous phase was extracted with DCM (3 × 30 mL). The organic phases were collected and rinsed with 1 M HCl (2 × 30 mL), dried with dried MgSO_4_, and filtered. DCM was removed under reduced pressure, and an orange-brown oil was obtained. The residue was purified by silica gel column chromatography (pentane: ethyl acetate (7:3)) and L″ was obtained as a pale green-yellow solid. Yield: 80%.^1^H NMR (400 MHz, DMSO-*d*_6_) δ: 10.23 (s, 1H), 7.31–7.23 (m, 2H), 6.91 (t, *J* = 7.9 Hz, 1H), 4.20–4.13 (m, 2H), 3.73–3.66 (m, 2H), 3.32 (s, 3H). ^13^C NMR (101 MHz, DMSO-*d*_6_) δ: 192.70, 150.88, 147.50, 122.49, 121.05, 119.28, 119.25, 70.26, 68.33, 58.18. ESI-MS calculated *m*/*z* for C_10_H_12_O_4_ + H^+^ ([M + H]^+^) = 197.1, found 197.4, ESI-MS calculated *m*/*z* for C_10_H_12_O_4_ + Na^+^ ([M + Na]^+^) = 219.1, found 219.0.

### Synthesis of Ligand H_2_L

The ligand was synthesized according to the literature procedure.^67^ L″ (0.2 g, 1.0 equiv, 1.0 mmol) was dissolved in ethanol (EtOH) (3 mL) and ethylenediamine (34 μL, 0.5 equiv, 0.05 mmol) was added dropwise. The solution was stirred at room temperature for 2 h. The pale green-yellow solution directly turned dark yellow after the addition of ethylenediamine. After 1 h, a yellow precipitate appeared. It was filtered, washed with cold EtOH (10 mL), and dried under vacuum overnight using a Schlenk line. Yield: 0.25 g, 60%. ^1^H NMR (400 MHz, DMSO-*d*_6_) δ: 13.58 (s, 2H), 8.57 (s, 2H), 7.02 (ddd, *J* = 7.9, 5.0, 1.5 Hz, 4H), 6.77 (t, *J* = 7.8 Hz, 2H), 4.11–4.05 (m, 4H), 3.93 (s, 4H), 3.68–3.61 (m, 4H), 3.30 (s, 6H). ^13^C NMR (101 MHz, DMSO-*d*_6_) δ: 167.16, 151.76, 147.05, 123.61, 118.47, 117.74, 116.51, 70.44, 67.94, 58.36, 58.18. ESI-MS calculated *m*/*z* for C_22_H_28_N_2_O_6_ + H^+^ ([M + H]^+^) = 417.2, found 417.1, and ESI-MS calculated *m*/*z* for C_22_H_28_N_2_O_6_ + Na^+^ ([M + Na]^+^) = 439.2, found 439.0.

### Synthesis of the Salen Ligand

The ligand was synthesized according to the literature procedure.^67^ Salicylaldehyde (2.0 g, 1.0 equiv, 16.38 mmol) was dissolved in EtOH (50 mL). Ethylenediamine (547 μL, 0.5 equiv, 8.19 mmol) was added dropwise. The solution was stirred at RT for 2 h. The solution immediately turned dark yellow after the addition of ethylenediamine. After 1 h, a yellow precipitate appeared. It was filtered and dried under a vacuum using a Schlenk line overnight. Yield: 1.77 g, 40.3%. ^1^H NMR (400 MHz, DMSO-*d*_6_) δ: 13.37 (s, 1H), 8.60 (s, 1H), 7.43 (dd, *J* = 7.7, 1.7 Hz, 1H), 7.32 (ddd, *J* = 8.3, 7.3, 1.7 Hz, 1H), 6.93–6.83 (m, 2H), 3.93 (s, 2H). ^13^C NMR (101 MHz, DMSO-*d*_6_) δ: 167.41, 161.03, 132.83, 132.13, 119.06, 119.03, 116.93, 59.22.

### Synthesis of [CuL­(H_2_O)­(CH_3_OH)] “LCu” (**1**)

H_2_L (0.50 g, 1.0 eq., 1.2 mmol) was dissolved in methanol (MeOH) (5 mL) at RT. Cu­(OAc)_2_·H_2_O (0.24 g, 1.0 eq., 1.2 mmol), previously dissolved in MeOH (1 mL), was added. The solution turned dark green immediately. The solution was stirred at RT for 2 h. The mother liquor was stored in a vial with an unsealed cap to allow the solvent to evaporate slowly over several days, affording crystals suitable for single-crystal X-ray diffraction. Dark blue prism-like single crystals suitable for X-ray crystallographic analysis were obtained. ESI-MS calculated *m*/*z* for C_22_H_26_N_2_O_6_Cu + H^+^ ([M + H]^+^) = 478.1, found 477.9.

### Synthesis of [NiL­(H_2_O)] “LNi” (**2**)

The same procedure was followed using Ni­(OAc)_2_·4H_2_O instead of Cu­(OAc)_2_·H_2_O. The solution turned red immediately after the addition of Ni­(OAc)_2_·4H_2_O. The mother liquor was stored in a vial with an unsealed cap to allow the solvent to evaporate slowly over several days, affording crystals suitable for single-crystal X-ray diffraction. Orange needle-like single crystals suitable for X-ray crystallographic analysis were obtained. ESI-MS calculated *m*/*z* for C_22_H_26_N_2_O_6_Ni + Na^+^ ([M + Na]^+^) = 495.1, found 495.1.

### Synthesis of [CuCaL­(NO_3_)_2_] “LCuCa” (**3**)

H_2_L (0.50 g, 1.0 eq., 1.2 mmol) was dissolved in MeOH (5 mL) at RT. Cu­(NO_3_)_2_··3H_2_O (0.29 g, 1.0 eq., 1.2 mmol), previously dissolved in MeOH (2 mL), was added. The solution was stirred for 2 h at RT. A solution of Ca­(NO_3_)_2_·4H_2_O (0.28 g, 1 eq., 1.2 mmol) in MeOH (2 mL) was poured inside the solution and stirred for 2 h at RT. The solution became red immediately. The addition of ether afforded the precipitation of a red powder. The mother liquor was stored in a vial with an unsealed cap to allow the solvent to evaporate slowly over several days, affording crystals suitable for single-crystal X-ray diffraction. Yield: 73%. Reddish violet prism-like single crystals suitable for X-ray crystallographic analysis were obtained. ESI-MS calculated *m*/*z* for [C_22_H_26_N_3_O_9_CuCa]^+^ = 579.0, found 578.7.

### Synthesis of [CuSrL­(NO_3_)_2_] “LCuSr” (**4**), [CuBaL­(NO_3_)_2_]­(H_2_O) “LCuBa” (**5**), [NiCaL­(NO_3_)_2_] “LNiCa” (**6**), [NiSrL­(NO_3_)_2_] “LNiSr” (**7**), and [NiBaL­(NO_3_)_2_] “LNiBa” (**8**)

For complexes **4** and **5**, the same procedure was followed by using Sr­(NO_3_)_2_ and Ba­(NO_3_)_2_, respectively. A mixture of MeOH/H_2_O (1:1) (2 mL) was used to solubilize the salts. After addition of the alkaline earth metal salt solutions, the mixture immediately turned red-purple and purple for **4** and **5**, respectively. For complexes **6**, **7**, **8**, the same procedure was followed using Ni­(NO_3_)_2_·6H_2_O instead of Cu­(NO_3_)_2_·3H_2_O and 1 eq. of the appropriate alkaline earth metal salt. After addition of Ni­(NO_3_)_2_·6H_2_O to the H_2_L ligand solution, it turned red immediately. However, no change in color was observed upon further addition of the alkaline earth metal solutions. Data for **4**: Yield: 83%. Violet needle-like single crystals suitable for X-ray crystallographic analysis were obtained. ESI-MS calculated *m*/*z* for [C_22_H_26_N_3_O_9_CuSr]^+^ = 627.0, found 626.6. Data for **5**: Yield: 83%. Yellow plate-like single crystals suitable for X-ray crystallographic analysis were obtained. ESI-MS calculated *m*/*z* for [C_22_H_26_N_3_O_9_CuBa]^+^ = 677.0, found 676.7. Data for **6**: Yield: 73%. Red plate-like single crystals suitable for X-ray crystallographic analysis were obtained. ESI-MS calculated *m*/*z* for [C_22_H_26_N_3_O_9_NiCa]^+^ = 574.1, found 574.1. Data for **7**: Yield: 67%. Red needle-like single crystals suitable for X-ray crystallographic analysis were obtained. ESI-MS calculated *m*/*z* for [C_22_H_26_N_3_O_9_NiSr]^+^ = 622.0, found 622.0. Data for **8**: Yield: 68%. Red hexagonal prism-like single crystals suitable for X-ray crystallographic analysis were obtained. ESI-MS calculated *m*/*z* for [C_22_H_26_N_3_O_9_NiBa]^+^ = 672.0, found 671.9.

### Solubility Test

Each heterobimetallic complex (10 mg) was added to different solvents (MeOH, EtOH, H_2_O, pentane, THF, toluene, dimethylformamide (DMF), DMSO, acetone (ACE), acetonitrile (ACN), chloroform (CH_3_Cl), and DCM). After shaking for a few seconds, observations were made to see if the complexes were completely dissolved, formed a suspension, or stayed as a solid precipitate.

### Agar Diffusion Test (Kirby–Bauer Antibiotic Testing)

An overnight culture of *S. aureus* (113 wt)^72^ in Müller–Hinton (MH) broth (10 mL) was diluted to a concentration of 1 × 10^5^ CFU/mL using the McFarland standard. The diluted culture (100 μL) was spread on agar plates. Pellets of the compounds were laid down on the agar before being incubated at 37 °C for 24 h. The next day, the diameter of the inhibition zone was measured by using a ruler. To obtain the inhibition zone radius, the pellet diameter was deducted before being divided by two.

## Results and Discussion

### Synthesis of the Ligand H_2_L

The ligand H_2_L was formed according to the literature.^67^ The precursor L″ is produced by the functionalization of 2,3-dihydroxybenzaldehyde with a glycol chain in *meta*-position via the Williamson ether synthesis.^73^ The H_2_L ligand is formed via the condensation reaction of L″ and ethylenediamine (Scheme 1). The ligand was characterized by ^1^H and ^13^C NMR, ESI-MS spectrometry, and powder X-ray diffraction.

**Scheme 1:**
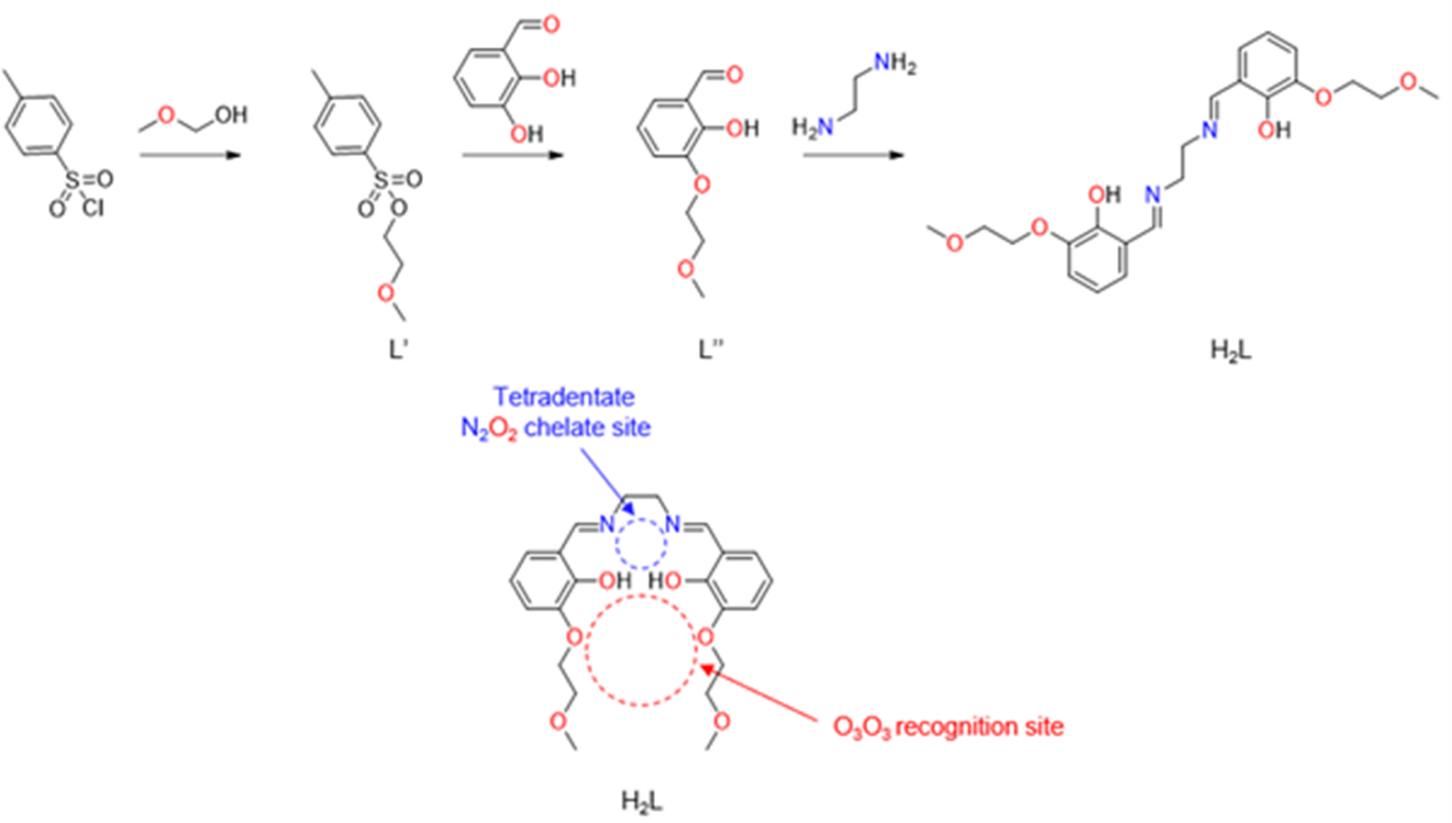
General Synthesis Pathway of H_2_L and Its Potential Coordination Sites

This ligand possesses a tetradentate N_2_O_2_ chelate site formed by two azomethine groups and two phenol rings. A first metal ion can be coordinated by the N_2_O_2_ site upon deprotonation of the hydroxide groups of the phenol rings. This complexation rearranges the ligand from a stretched-out shape into an Ω-shape, creating thus a second O_3_O_3_ recognition site, formed by the two O atoms of the phenolates and the four O atoms of the glycol chains, enabling the coordination of a second metal ion.^74^

### Crystallography

To verify whether the O_3_O_3_ recognition site is available after coordination of the first metal ion, single crystal X-ray analyses were performed on monometallic complexes. Furthermore, as alkaline earth metal ions do not have a coordination geometry preference,^75^ single crystals were grown to better understand their coordination by the H_2_L ligand. The different complexes were crystallized by slow evaporation of the mother liquor, affording suitable crystals for single X-ray analysis. The crystallographic data of the eight crystal structures are detailed in Table 1.

**Table 1:** Crystal Data and Structure Refinement for Complexes **1–8**

identification code	LCu (**1**)	LNi (**2**)	LCuCa (**3**)	LCuSr (**4**)	LCuBa (**5**)	LNiCa (**6**)	LNiSr (**7**)	LNiBa (**8**)
CCDC number	CCDC-2463279	CCDC-2463280	CCDC-2463281	CCDC-2463282	CCDC-2463283	CCDC-2463284	CCDC-2463285	CCDC-2463286
empirical formula	C_23_H_32_CuN_2_O_8_	C_22_H_28_N_2_NiO_7_	C_22_H_26_CaCuN_4_O_12_	C_22_H_26_CuN_4_O_12_Sr	C_22_H_28_BaCuN_4_O_13_	C_22_H_26_CaN_4_NiO_12_	C_22_H_26_N_4_NiO_12_Sr	C_22_H_26_BaN_4_NiO_12_
formula weight	528.04	491.17	642.09	689.63	757.36	637.26	684.80	734.52
temperature/K	250(2)	250(2)	250(2)	250(2)	250(2)	250(2)	250(2)	250(2)
crystal system	monoclinic	monoclinic	orthorhombic	orthorhombic	monoclinic	monoclinic	orthorhombic	orthorhombic
space group	*C*2/*c*	*C*2/*c*	*Pbca*	*Pbca*	*P*2_1_/*n*	*P*2_1_/*c*	*Pbca*	*Pbca*
*a*/Å	26.3789(7)	18.7924(4)	13.63820(10)	13.8605(3)	9.0487(2)	15.8439(11)	13.7945(2)	13.8849(4)
*b*/Å	8.07900(10)	20.1880(4)	28.6812(3)	13.6247(3)	27.8334(5)	13.9063(7)	13.3697(2)	13.4226(4)
*c*/Å	23.2287(5)	13.8617(3)	13.7101(2)	28.8461(6)	10.9684(3)	12.4247(7)	29.3373(4)	29.6517(9)
β/°	102.087(2)	122.0880(10)	90	90	96.964(2)	104.787(5)	90	90
volume/Å^3^	4840.64(18)	4455.48(17)	5362.84(10)	5447.4(2)	2742.08(11)	2646.9(3)	5410.63(13)	5526.2(3)
*Z*	8	8	8	8	4	4	8	8
ρ_calc_g/cm^3^	1.449	1.464	1.591	1.682	1.835	1.599	1.681	1.766
μ/mm^–1^	1.708	1.657	3.447	4.157	1.198	0.521	4.080	12.463
*F*(000)	2216.0	2064.0	2648.0	2792.0	1508.0	1320.0	2784.0	2928.0
crystal size/mm^3^	0.92 × 0.48 × 0.2	0.66 × 0.353 × 0.2	0.24 × 0.12 × 0.06	0.64 × 0.32 × 0.1	0.42 × 0.273 × 0.1	0.99 × 0.56 × 0.13	0.66 × 0.267 × 0.05	0.95 × 0.51 × 0.08
Radiation	Cu Kα (λ = 1.54186)	Cu Kα (λ = 1.54186)	Cu Kα (λ = 1.54186)	Cu Kα (λ = 1.54186)	Ag Kα (λ = 0.56083)	Ag Kα (λ = 0.56083)	Cu Kα (λ = 1.54186)	Cu Kα (λ = 1.54186)
2Θ range for data collection/°	13.472 to 133.834	7.892 to 137.478	8.924 to 135.506	8.848 to 136.322	4.258 to 51.948	4.446 to 53.182	9.694 to 136.69	13.528 to 133.606
index ranges	–30 ≤ *h* ≤ 31, –9 ≤ *k* ≤ 6, –27 ≤ *l* ≤ 25	–18 ≤ *h* ≤ 22, –23 ≤ *k* ≤ 22, –15 ≤ *l* ≤ 11	–5 ≤ *h* ≤ 15, –32 ≤ *k* ≤ 32, –15 ≤ *l* ≤ 15	–16 ≤ *h* ≤ 15, –7 ≤ *k* ≤ 15, –32 ≤ *l* ≤ 34	–13 ≤ *h* ≤ 14, –43 ≤ *k* ≤ 43, –16 ≤ *l* ≤ 17	–23 ≤ *h* ≤ 25, –21 ≤ *k* ≤ 21, –19 ≤ *l* ≤ 17	–16 ≤ *h* ≤ 16, –11 ≤ *k* ≤ 15, –35 ≤ *l* ≤ 34	–16 ≤ *h* ≤ 14, –9 ≤ *k* ≤ 15, –33 ≤ *l* ≤ 34
reflections collected	37,828	30,778	55,885	98,753	67,927	39,030	51,419	34,613
independent reflections	4220 [*R*_int_ = 0.0756, *R*_sigma_ = 0.0338]	4017 [*R*_int_ = 0.0681, *R*_sigma_ = 0.0351]	4545 [*R*_int_ = 0.0431, *R*_sigma_ = 0.0187]	4830 [*R*_int_ = 0.0385, *R*_sigma_ = 0.0123]	9862 [*R*_int_ = 0.0566, *R*_sigma_ = 0.0292]	9550 [*R*_int_ = 0.0842, *R*_sigma_ = 0.0875]	4885 [*R*_int_ = 0.0701, *R*_sigma_ = 0.0316]	4729 [*R*_int_ = 0.1103, *R*_sigma_ = 0.0514]
data/restraints/parameters	4220/4/339	4017/43/327	4545/2/373	4830/62/414	9862/0/375	9550/22/363	4885/34/408	4729/46/369
goodness-of-fit on F^2^	1.062	1.058	1.098	1.031	1.052	0.913	1.089	1.046
final R indexes [*I* ≥ 2σ (*I*)]	*R*_1_ = 0.0556, w*R*_2_ = 0.1501	*R*_1_ = 0.0436, w*R*_2_ = 0.1111	*R*_1_ = 0.0477, w*R*_2_ = 0.1429	*R*_1_ = 0.0416, w*R*_2_ = 0.1139	*R*_1_ = 0.0261, w*R*_2_ = 0.0651	*R*_1_ = 0.0420, w*R*_2_ = 0.0871	*R*_1_ = 0.0466, w*R*_2_ = 0.1229	*R*_1_ = 0.0792, w*R*_2_ = 0.2229
final *R* indexes [all data]	*R*_1_ = 0.0558, w*R*_2_ = 0.1505	*R*_1_ = 0.0449, w*R*_2_ = 0.1120	*R*_1_ = 0.0519, w*R*_2_ = 0.1460	*R*_1_ = 0.0441, w*R*_2_ = 0.1173	*R*_1_ = 0.0368, w*R*_2_ = 0.0690	*R*_1_ = 0.1042, w*R*_2_ = 0.1004	*R*_1_ = 0.0518, w*R*_2_ = 0.1255	*R*_1_ = 0.0851, w*R*_2_ = 0.2344
largest diff. peak/hole/e Å^–3^	1.10/–0.69	0.35/–0.48	0.84/–0.44	0.92/–0.65	0.48/–0.56	0.42/–0.30	0.69/–0.55	1.80/–1.00

For the monometallic complexes **1** and **2**, a 1:1 (ligand/metal) stoichiometry was obtained. The transition metal ion (Cu^2+^ and Ni^2+^, respectively) is coordinated to the tetradentate N_2_O_2_ chelate site in a quasi-perfect square planar geometry with angle sum values close to 360°. For both, each H atom of the water molecule “captured” by four O atoms of the O_3_O_3_-site is involved in two H-bonds with the two O-Atoms of the aromatic rings, namely, O1 and O3, respectively, O2 and O4 (Figure 1). The LCu complex has an additional methanol molecule involved in hydrogen bonding with the water molecule. The bond valence sums^76^ (BVS) of Cu^2+^ and Ni^2+^ have values of 2.23 and 2.51, respectively. The angles between the two planes of the aromatic rings are 17.87° and 5.75° for **1** and **2**, respectively.

**Figure 1:**
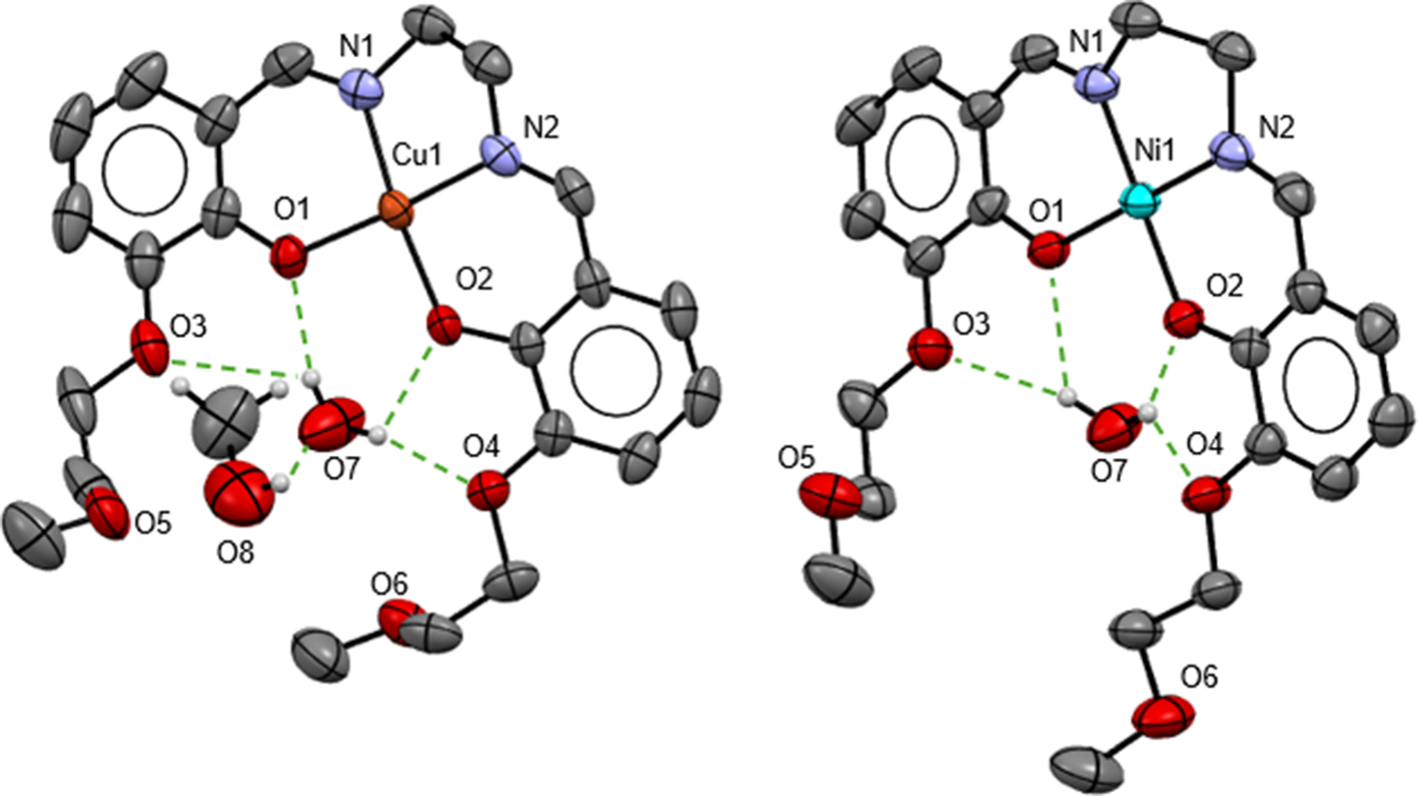
Structure of the asymmetric unit of LCu (**1**) (left) and LNi (**2**) (right). All H atoms, except H atoms of water and methanol molecules, are omitted for clarity.

**Table 2:** MMOs Obtained after Thermal Treatment at 1000 °C in Air for One H

single-source precursor(s)	(mixed) metal oxides obtained	temperature (°C)
LCuCa (**3**)	Ca_2_CuO_3_ (25%)	1000
	CuO (50%)	
	CaO (25%)	
LCuCa (**3**)	CaO (54%)	800
	CuO (46%)	
LCuSr (**4**)	SrCuO_2_ (93%)	1000
	Sr_2_CuO_3_ (7%)	
LCuBa (**5**)	BaCO_3_ (77%)	1000
	CuO (23%)	
LNiCa (**6**)	NiO (60%)	1000
	CaO (40%)	
LNiSr (**7**)	Ni_2.5_Sr_4_O_9_ (31%)	1000
	Ni_6.64_Sr_9_O_21_ (25%)	
	NiO (44%)	
LNiBa (**8**)	Ni_5_Ba_6_O_15_, (91%)	1000
	NiO (9%)	
LCuCa (**3**) and LCuSr (**4**)	Ca_0.38_Sr_0.62_CuO_2_ (72%)	1000
	Ca_1.50_Sr_0.50_CuO_3_ (28%)	
LCuCa (**3**) and LNiSr (**7**)	CaSrCuO_3_ (78%)	1000
	NiO (22%)	
LCuSr (**4**) and LNiCa (**6**)	CaSrCuO_3_ (81%)	1000
	NiO (19%)	

Regarding the heterometallic complexes, they all have a 1:1:1 (L/M_1_/M_2_) stoichiometry. As the monometallic complexes **1** and **2**, the Cu^2+^ and Ni^2+^ ions for **3**, **4**, **5** and **6**, **7**, and **8**, respectively, are coordinated by the N_2_O_2_ tetradentate chelate site in a quasi-perfect square planar geometry with angle sum values close to 360° (Figure 2). The alkaline earth metal ions are coordinated by the six O atoms of the ligand, except for the LNiCa (**6**) complex, in which the calcium ion is only coordinated by five O atoms, while the O5 of the ligand is pointing outward with respect to the binding cavity. The Ca^2+^ ion has shorter bond lengths with the O atoms in the LNiCa (**6**) complex compared to the LCuCa (**5**) complex (Table S1). Therefore, the bonds have a higher electrostatic valence and can compensate the +2 charge of the calcium ion without the coordination of the O5 atom.^77^ Furthermore, we reported an homologous copper­(II)–calcium salophen complex that exhibits analogous coordination mode as the LNiCa complex with similar bond lengths and angles.^71^ Therefore, the O5 atom seems to act as a “joker” coordination site. Similar observations were previously reported by our research group concerning a different calcium complex, crystallizing in two different isomeric forms, in which one O-donor atom of a given ligand was once coordinated, once not.^63^ Their BVS were calculated considering all the contacts with a minimum bond valence contribution of 0.08 (=0.04 × cation valence), leading to values of 2.01, 2.24, 2.38, 2.26, 2.29 and 2.58, for **3**, **4**, **5**, **6**, **7**, and **8**, respectively. For all the heterobimetallic complexes, the second metal ion is out of the plane formed by the four O atoms (O1, O2, O3, and O4) by approximately 0.09, 0.08, 0.42, 0.35, 0.05, and 0.16 Å for **3**, **4**, **5**, **6**, **7**, and **8**, respectively, while the angles between the two planes of the aromatic rings are 16.7°, 16.0°, 8.9°, 17.8°, 16.3°, and 15.0°, respectively. Concerning the packing, the six crystal structures are discrete, i.e., no remarkable H-bonds or π-stacking in the unit cells were observed. Only the crystal structure of LCuBa **(5)** contains one water molecule O13 per asymmetric unit, which, together with O13′, acts as bridge, thereby connecting two complexes via H-bonding with two O atoms (O11 and O12) of one nitrate group with a graph set R_4_^4^(12)^76^ (Figure 3). An inversion center is found in the geometrical middle of the ring formed by the two water molecules and two nitrate anions (Figure 3).

**Figure 2:**
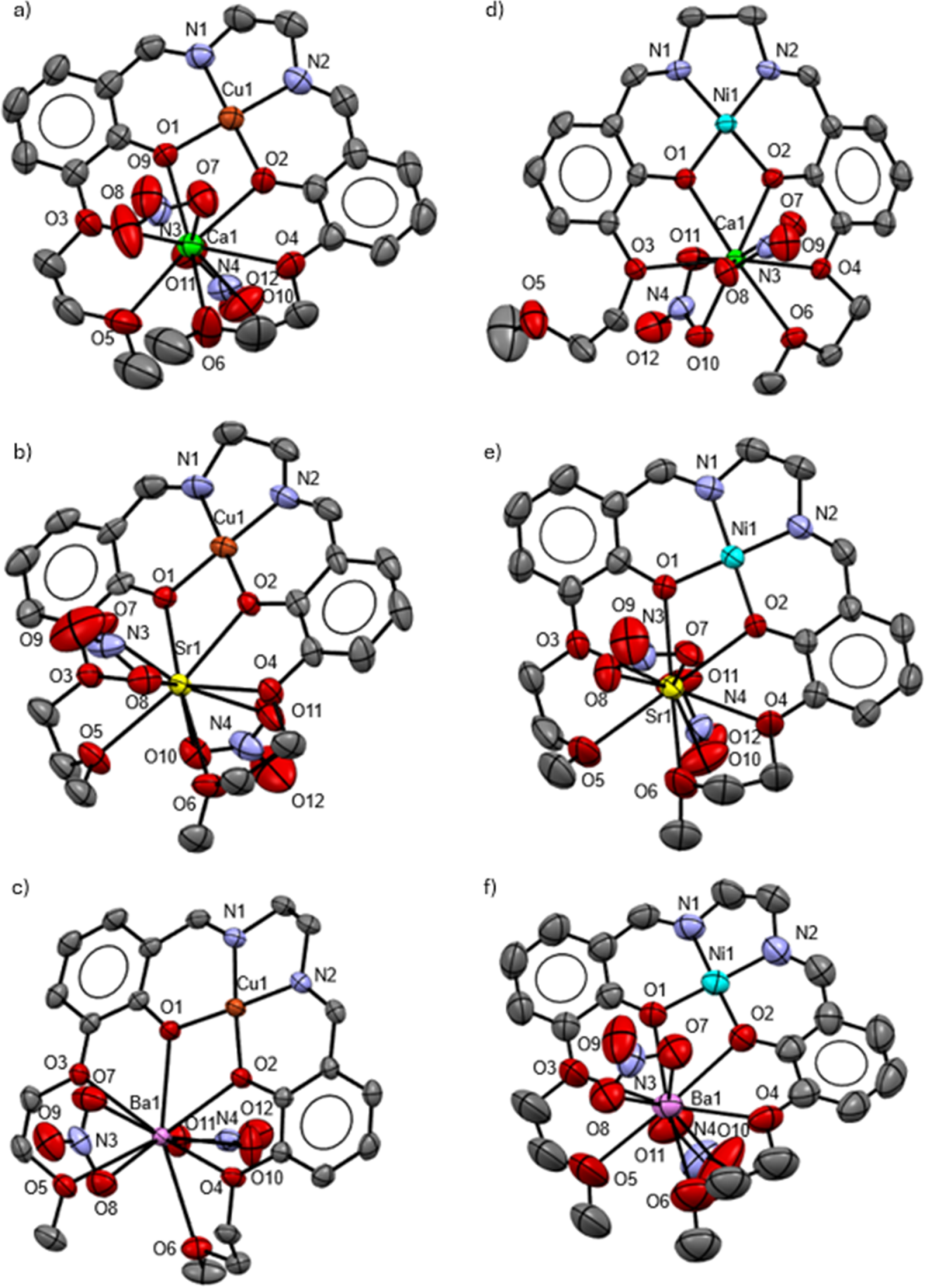
Structure of the asymmetric units of (a) LCuCa (**3**), (b) LCuSr (**4**), (c) LCuBa (**5**), (d) LNiCa (**6**), (e) LNiSr (**7**), and (f) LNiBa (**8**). All H atoms and solvent molecules are omitted for clarity.

**Figure 3:**
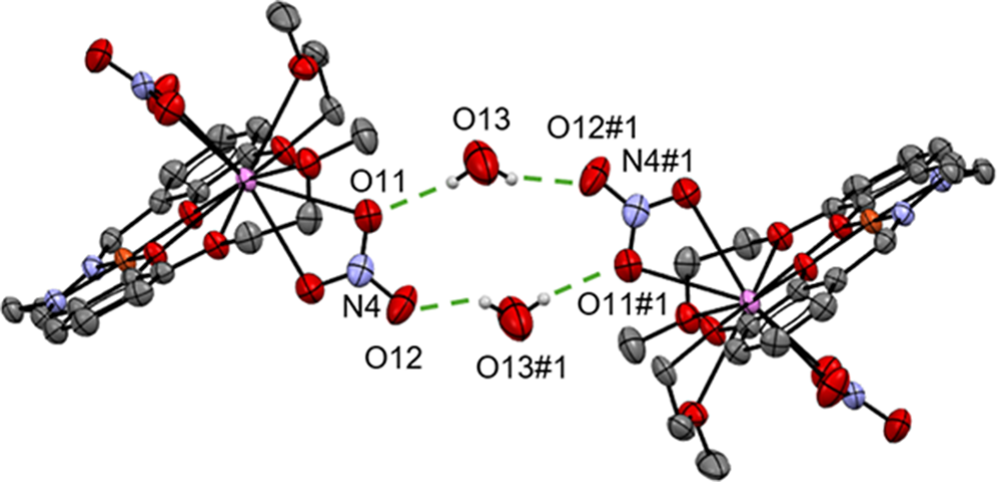
Packing of LCuBa (**5**) showing the formation of a dimer through H-bonds (#1 (2 – *x*, 1 – *y*, −*z*)).

### Single-Source Precursor

Thermogravimetric analyses (TGA) were performed on each heterometallic complex from 25 °C until 1000 °C under air (50 mL/min) with an increase of 10 °C per minute (Table 2). After calcination, the compositions of the obtained powders were analyzed by powder X-ray diffraction (XRPD).

In a general manner, the decomposition of the complexes begins with a first and major step around 320–330 °C corresponding to the partial or total decomposition of the organic part. LNiBa is the only complex showing a first weight loss at around 250 °C. This is followed by one or multiple steps for the complete decomposition of the organic part, including the nitrate moieties. As a general observation, the larger the alkaline earth metal ion, the higher the temperature of total decomposition. For calcium-based complexes, the complexes are totally decomposed at 700 °C for LCuCa (**3**) and 680 °C for LNiCa (**6**). For the strontium-based complexes, LCuSr (**4**) and LNiSr (**7**), the complete decomposition occurred at 830 and 920 °C, respectively. For LCuBa (**5**), the organic part does not seem to be completely decomposed at 1000 °C and for LNiBa (**8**), the total decomposition occurs around 930 °C (Figures S19–S24).

To test the ability of the six heterometallic complexes as single-source precursors, they were all thermally treated under air at 1000 °C during 1 h. The sample was allowed to cool to room temperature after switching off the oven. For LCuCa, the XRPD pattern showed the formation of a mixed metal oxide, Ca_2_CuO_3_ (25%) and two simple metal oxides CuO (50%) and CaO (25%) (Figure S9). According to the literature,^78^ Ca_2_CuO_3_ was synthesized by pyrolysis at 960 °C for 10 h in air using CaCO_3_ and CuO as starting reagents. Another way to synthesize Ca_2_CuO_3_ according to Hassani et al.^79^ is to dissolve Ca­(NO_3_)_2_·4H_2_O and Cu­(OAc)_2_·H_2_O in water and heat it to form a gel. The gel was calcined for 3 h in air at 900 °C. When the LCuCa (**3**) complex is calcined using milder conditions, heating only to 800 °C, only the simple metal oxides, CaO (54%) and CuO (46%) in a roughly 1:1 ratio, were obtained (Figure S10). For LCuSr, two mixed metal oxides, SrCuO_2_ (93%) and Sr_2_CuO_3_ (7%), were obtained (Figure S11). Sachith et al. used the coprecipitation method followed by a sonochemical process to synthesize SrCuO_2_.^80^ According to the literature,^81^ Sr_2_CuO_3_ was synthesized by dissolving Cu­(NO_3_)_2_·3H_2_O and Sr­(NO_3_)_2_·H_2_O in a diethylene glycol monobutyl ether to form a xerogel, heated at different temperatures (400, 700, and 950 °C). Sr_2_CuO_3_ was also synthesized using the traditional solid state method by mixing SrO and CuO for 1 h and sintering it to 980 °C for 20 h at air.^82^ The second step was to heat it again to 980 °C for 10 h with full grindings. For LCuBa, witherite (BaCO_3_) (77%) and simple CuO (23%) were obtained (Figure S12). The unbalanced percentage could be due to the presence of copper in the amorphous phase. The presence of carbonate is explained by the incomplete decomposition of the organic part, as observed by the TGA measurement (Figure S21). For LNiCa, only the simple metal oxides, NiO (60%) and CaO (40%), were observable in the XRPD pattern (Figure S13). For LNiSr, two mixed metal oxides, Ni_2.5_Sr_4_O_9_ (31%), and Ni_6.64_Sr_9_O_21_ (25%), were formed as well as the simple metal oxide NiO (44%) (Figure S14). Strunk et al. formed Ni_2.5_Sr_4_O_9_ by heating a mixture of SrCO_3_ and NiO at 1000 °C for 24 h.^83^ After calculation of the Coulomb terms of the lattice energy, the most accurate model was where all the nickel ions in Ni_2.5_Sr_4_O_9_ have an oxidation state of 4^+^.^84^ According to the literature, Ni_6.64_Sr_9_O_21_ was formed by heating to 880 °C a mixture of Sr­(OH)_2_·8H_2_O, NiO and KOH, followed by a soaking of 2 h.^84^ Concerning the oxidation state of nickel in this MMO, no models were perfectly fitting.^84^ For LNiBa, a mixed metal oxide, Ni_5_Ba_6_O_15_, (91%) and simple metal oxide NiO (9%) were obtained (Figure S15). According to the literature,^85^ Ni_5_Ba_6_O_15_ was obtained by heating a mixture of NiO, BaCO_3_, and KOH for 10 min at 750 °C. After cooling down to RT, the mixture was reheated for a further 10 min at 1100 °C. For the oxidation state of nickel ions in Ni_5_Ba_6_O_15_, different models were proposed: (1) an ordered distribution of Ni^4+^ (low spin), Ni^3+^ (low spin), and Ni^2+^ and (2) an ordered structure of Ni^4+^ (low spin) and Ni^2+^.^84^

Further investigations were conducted by mixing and thermally treating the powder of two heterometallic complexes at 1000 °C for the potential formation of mixed metal oxides containing three or four different metal ions. LCuCa and LCuSr complexes were mixed in a 1:1 stoichiometry and heated under air to 1000 °C for 1 h. The XRPD pattern showed the formation of two mixed metal oxides, Ca_0.38_Sr_0.62_CuO_2_ (72%), and Ca_1.50_Sr_0.50_CuO_3_ (28%), containing the three metal ions with different ratios (Figure S16). According to the literature,^86^ Ca_0.38_Sr_0.62_CuO_2_ was obtained by heating to 1045 °C within 5 h a mixture of SrCO_3_, CaCO_3_, and CuO. Lines et al. synthesized Ca_1.50_Sr_0.50_CuO_3_ by heating to 870 °C a mixture of CuO, CaCO_3_, and SrCO_3_.^87^ The same experiment was repeated this time using the LNiSr complex instead of the LCuSr complex to potentially form MMO with four different metal ions. After thermal treatment, the formation of MMO, CaSrCuO_3_ (78%), and simple metal oxide, NiO (22%) was observed by XRPD (Figure S17). To see if the assortment of the metal ions within the ligand is important for the formation of MMO, the experiment was repeated mixing the LNiCa complex and LCuSr complex. After thermal treatment, the same results were obtained, i.e., the formation of CaSrCuO_3_ (81%) and NiO (19%) metal oxide (Figure S18). In the literature, CaSrCuO_3_ was synthesized using the same procedure as Ca_1.50_Sr_0.50_CuO_3_ with the adapted stoichiometry.^87^ The CaSrCuO_3_ oxide has an orthorhombic *Immm* space group.^87^ The different sizes of the Ca^2+^ and Sr^2+^ cations can induce that Cu fits better than Ni, as NiO prefers cubic structures.^88^

The morphology of the obtained MMOs was studied by SEM and revealed a sponge-like structure for all the samples (Figures 4 and S25–S31), likely resulting from the loss of gaseous substances, such as water and CO_2_.

**Figure 4:**
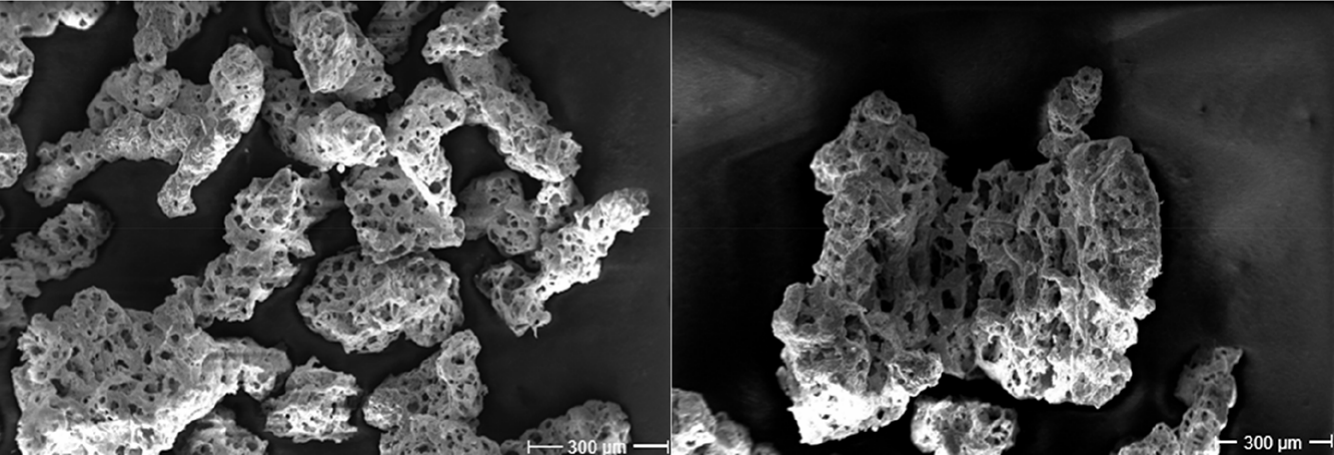
SEM image of LCuSr (**5**) (left) and mixture of LCuCa (**4**) and LCuSr (**5**) (right) after annealing at 1000 °C in air for 1 h.

In comparison with the listed literature procedures, the calcination temperature is approximately the same, whereas the time of calcination is shorter in our study. A longer time of calcination would have probably favored the formation of only one MMO for the complexes LCuSr, LNiSr, and the mixture LCuCa/LCuSr as the obtained MMOs are not formed at the same temperature. The utilization of SSPs based on salen-type heterobimetallic complexes does not seem to have advantages compared to the classical methods found in the literature. Indeed, when the complexes are thermally treated under air to 600 °C for 1 h, the XRPD analyses revealed the formation of carbonate salts for the alkaline earth metals and simple oxides for the transition metals (Figures S19–S21), which would amount to directly using the corresponding salts as starting materials.

The solubility of the complexes was tested in different solvents and showed good solubility in MeOH, DMF, and DMSO. Therefore, other techniques based on solutions for the formation of mixed metal oxides can be explored.

### Antibacterial Properties

As copper is known to possess antibacterial properties^89^ and calcium and strontium are biocompatible,^90,91^ and the integration of barium in different biomaterials is tested,^92–94^ Kirby–Bauer assays of the copper-based complexes (**1**), (**3**), (**4**), and **(5)** as well as the ligand H_2_L and the salen ligand were carried out on*S. aureus*. The powders of the different heterobimetallic complexes and the ligand were weighed, pressed into individual pellets, and placed on an agar plate previously seeded with*S. aureus*­(1 × 10^5^ CFU/mL). After incubation at 37 °C for 24 h, the zone of inhibition (ZOI) was measured for each sample.

The Kirby–Bauer assays (Figure 5) showed that the ligand, H_2_L, diffused well into the agar plate and showed a ZOI of 10.0 mm (mm). When the ligand is coordinated to Cu^2+^, the ZOI increases by 11.5 mm. However, for heterobimetallic complexes **(3)**, **(4)**, and **(5)**, the ZOI decreases drastically. The LCuSr **(4)** and LCuBa **(5)** complexes both have a ZOI of 5.0 mm, whereas the ZOI of the LCuCa **(3)** complex has a value of 3.5 mm (Table 3).

**Figure 5:**
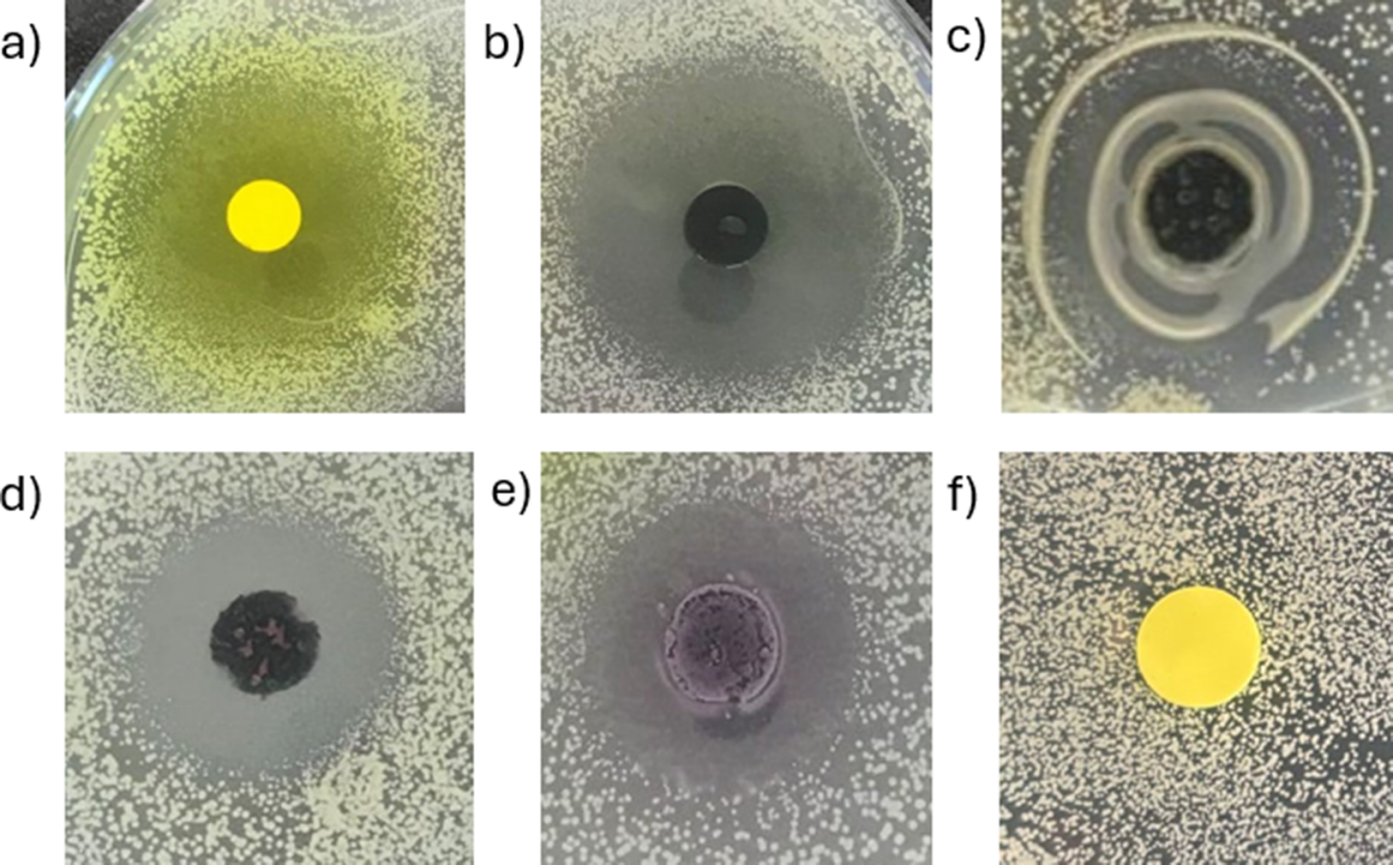
Kirby–Bauer assays of (a) the ligand H_2_L (b) LCu (**1**), (c) LCuCa (**3**), (d) LCuSr (**4**), (e) LCuBa (**5**), and (f) the salen ligand.

**Table 3:** ZOI, Weight and Molar Quantity of H_2_L and Its Mono- and Heterobimetallic Complexes

	H_2_L	LCu	LCuCa	LCuSr	LCuBa
ZOI [mm]	10.0	11.5	3.5	5.0	5.0
Weight [mg]	28	37	37	36	49
mmol	0.067	0.069	0.050	0.044	0.052
normalized ZOI [mm/mmol]	149.25	166.67	70.00	113.63	96.15

From a molar quantity perspective, the values of the H_2_L ligand and the LCu complex are higher than those of the heterobimetallic complexes as well as their normalized ZOI (Table 3). This factor can also be a contribution to their higher ZOI compared to that of the heterobimetallic complexes.

In order to better understand the role of the glycol chains, the same experiment was carried out with the salen ligand (formed by a condensation reaction between ethylenediamine and salicylaldehyde), for which no yellow coloration and ZOI were observed. The presence of glycol chains facilitates diffusion within the agar plate, demonstrated by the yellow coloration around the H_2_L pellet (Figure 5a). This also suggests that the glycol chains are involved in the antimicrobial effect, which is consistent with the literature.^95,96^ The studies on antimicrobial properties of glycols are limited but it is hypothesized to be related to an alteration of membrane function in bacteria and fungi.^97^ As the glycol chains of the heterobimetallic complexes are coordinated to the second metal ion (Ca^2+^, Sr^2+^ or Ba^2+^), they are no longer free, which could slow down their diffusion in the agar plate and thus reduce their antimicrobial effect and thus explain the smaller ZOI.

In order to gain an understanding of the effectiveness of the monometallic LCu complex, which showed the highest ZOI, a comparison with similar Schiff base complexes found in the literature was made (Table 4).

**Table 4:** ZOI of the Different Copper­(II) Complexes

	LCu (1)	Al-Shboul et al.^98^Cu(II)-complex	Salma et al.^99^ Cu(II)-complex	Abdalla et al.^100^Cu(II)-complex
bacterial strain	*S. aureus* (113 wt)	*S. aureus* (ATCC 29213)	*S. aureus*	*S. aureus*
ZOI [mm]	11.5	11.0	21.0	30.0

The monometallic LCu complex shows a comparable ZOI as reported by Al-Shboul et al. This is approximately two to three times less than the values obtained by Salma et al.^99^ and Abdalla et al.,^100^ respectively (Table 4). Compared with the literature, the LCu complex showed lower antibacterial activity. These differences could potentially be explained by the structure of the ligand and the metal coordination environment. Indeed, Salma et al.^99^ as well as Abdalla et al.^100^ selected the salen ligand as the organic part, whose antibacterial properties increased significantly when it is complexed with a metal ion.^101^ Furthermore, the addition of two imidazole entities in the axial position of the Cu^2+^ ion increases the ZOI by 9.0 mm (Table 4).^100^

## Conclusions

The assortments of different metal ions within one salen-type ligand, forming mono- and heterobimetallic complexes, were studied. The single X-ray crystal structure revealed 1:1 and 1:1:1 (L/M_1_:M_2_) stoichiometries with a similar coordination mode for all the complexes, except LNiCa. The properties of the six heterometallic complexes as SSPs were investigated. Four complexes, LCuCa, LCuSr, LNiSr, and LNiBa, showed the formation of mixed metal oxides when thermally treated at 1000 °C in air for 1 h. However, the presence of simple metal oxides, such as NiO or CuO, was also determined in the calcined powder. Further investigations, by mixing two powders, were performed. When **3** and **7** or **4** and **6** were mixed, the same MMOs were obtained with approximately the same percentage, meaning that the compartmentalization within the H_2_L does not have an influence. However, when the nickel ion is replaced by copper, it induces the formation of different MMOs indicating that the nature of the fourth metal ion has an influence. The good solubility of the six heterobimetallic complexes in MeOH, DMF, and DMSO offers the possibility of exploring other methods requiring precursors in solution for the formation of MMOs. Furthermore, the antibacterial effects of copper-based complexes were investigated. The ligand and the copper­(II) monometallic complex showed a higher ZOI compared with the heterobimetallic complexes, which is probably due to the presence of free glycol chains. The antimicrobial properties appear to be significantly influenced by the structure of the ligand and the metal coordination environment. In order to explore other potential applications, such as sensing, further studies on salen-type derivative complexes are ongoing in our research group.

## Supplementary Material



## Notes

Bond lengths and angles; lattice parameters; ^1^H and ^13^C NMR spectra; TGA spectra; XRPD patterns; SEM images; and ESI-MS spectra (PDF)
