# Supplementary material for: Schiff Base Heterometallic Complexes and Their Potential Applications
Source: Cryst Growth Des. 2026 Feb 9;26(4):1513–24. doi: 10.1021/acs.cgd.5c01024 (PMC12921694; doi:10.1021/acs.cgd.5c01024)
Supplement: Supplementary file 1 [file cg5c01024_si_001.pdf]

Supporting Information

Schiff Base heterometallic complexes and their potential applications

*Jocelyn Pradegan, Aurélien Crochet, and Katharina M. Fromm**

Department of Chemistry, University of Fribourg, Chemin du Musée 9, Fribourg, Switzerland

Contents

X-ray single crystal and powder diffraction....2

Thermogravimetric analysis (TGA) 14

SEM Images 16

^{1}H and ^{13}C NMR Spectra....19

ESI-MS Spectra 24

Single crystal and powder X-Ray diffraction

Table S1. Bond lengths in [Å].

	LCuCa (**3**)	LNiCa (**6**)
M_1-N_1	1.936(3)	1.8455(16)
M_1-N_2	1.921(3)	1.8317(16)
M_1-O_1	1.896(2)	1.8399(12)
M_1-O_2	1.901(2)	1.8513(13)
M_2-O_1	2.471(2)	2.3748(14)
M_2-O_2	2.483(2)	2.3780(13)
M_2-O_3	2.715(2)	2.6225(14)
M_2-O_4	2.712(3)	2.5380(14)
M_2-O_5	2.663(4)	-
M_2-O_6	2.575(3)	2.5170(13)
M_2-O_7	2.507(3)	2.4768(14)
M_2-O_8	2.503(3)	2.5270(15)
M_2-O_{10}	2.514(3)	2.4598(15)
M_2-O_{11}	2.558(3)	2.4711(14)

Table S2. Bond angles in [°].

	LCuCa (**3**)	LNiCa (**6**)
N_1-M_1-N_2	84.46(13)	86.36(8)
N_1-M_1-O_1	94.38(11)	94.49(7)
N_2-M_1-O_2	95.33(11)	95.26(7)
O_1-M_1-O_2	85.81(9)	83.88(6)
O_1-M_2-O_2	62.90(7)	62.54(4)
O_1-M_2-O_3	58.88(7)	62.49(4)
O_2-M_2-O_4	59.01(7)	63.84(4)
O_3-M_2-O_5	60.31(14)	-
O_4-M_2-O_6	59.96(9)	65.28(4)
O_5-M_2-O_6	67.46(17)	-

Table S3. Lattice parameters of the crystal structures and after Rietveld refinement.

	Lattice parameters	
	Crystal structures [Å]	Values obtained after Rietveld refinement [Å]
LCu (**1**)	a = 26.379<br>b = 8.079<br>c = 23.229	a = 27.049<br>b = 8.157<br>c = 23.675
LCuCa (**3**)	a = 13.638<br>b = 28.681<br>c = 13.710	a = 13.588<br>b = 28.687<br>c = 13.725
LCuSr (**4**)	a = 13.861<br>b = 13.625<br>c = 28.846	a = 13.870<br>b = 13.539<br>c = 28.931
LCuBa (**5**)	a = 9.049<br>b = 27.833	a = 9.041<br>b = 27.848

	c = 10.968	c = 10.993
LNiCa (**6**)	a = 15.844<br>b = 13.906<br>c = 12.425	a = 15.924<br>b = 13.997<br>c = 12.480
LNiSr (**7**)	a = 13.795<br>b = 13.370<br>c = 29.337	a = 13.790<br>b = 13.365<br>c = 29.294
LNiBa (**8**)	a = 13.885<br>b = 13.423<br>c = 29.652	a = 13.941<br>b = 13.334<br>c = 29.526

Table S4. Lattice parameters of mixed metal oxides (theoretical) and results obtained after Rietveld refinement (experimental).

	Lattice parameters								
	Oxide	Theoretical [Å]	Experimental [Å]	Oxide	Theoretical [Å]	Experimental [Å]	Oxide	Theoretical [Å]	Experimental [Å]
LCuCa (**3**)	Ca_2CuO_3	a = 12.239<br>b = 3.779<br>c =3.259	a = 12.211<br>b = 3.769<br>c =3.251	CuO	a = 4.684<br>b = 3.427<br>c =5.129	a = 4.677<br>b = 3.413<br>c =5.119	CaO	a = 4.805	a = 4.800
LCuCa (**3**)<br>(800 °C)	CuO	a = 4.684<br>b = 3.427<br>c =5.129	a = 4.681<br>b = 3.412<br>c =5.121	CaO	a = 4.805	a = 4.802			
LCuSr (**4**)	$SrCuO_2$	a = 3.565<br>b = 16.326<br>c =3.921	a = 3.564<br>b = 16.288<br>c = 3.903	Sr_2CuO_3	a = 12.688<br>b = 3.912<br>c = 3.485	a = 12.683<br>b = 3.905<br>c = 3.492			
LCuBa (**5**)	$BaCO_3$	a = 5.313<br>b = 8.896<br>c = 6.428	a = 5.297<br>b = 8.873<br>c = 6.432	CuO	a = 4.683<br>b = 3.420<br>c = 5.125	a = 4.673<br>b = 3.417<br>c = 5.115			
LNiCa (**6**)	CaO	a = 4.805	a = 4.799	NiO	a = 4.177	a = 4.176			
LNiSr (**7**)	$Ni_{6.64}Sr_9O_{21}$	a = 9.467<br>c = 35.870	a = 9.455<br>c = 35.687	$Ni_{2.50}Sr_4O_9$	a = 9.474<br>c = 7.802	a = 9.451<br>c = 7.775	NiO	a = 4.177	a = 4.168
LNiBa (**8**)	$Ni_5Ba_6O_{15}$	a = 9.889<br>c = 12.867	a = 9.886<br>c = 12.916	NiO	a = 4.177	a = 4.168			
LCuCa (**3**) &<br>LCuSr (**4**)	$Ca_{0.38}Sr_{0.62}CuO_2$	a = 3.321<br>b = 3.811<br>c = 12.360	a = 3.338<br>b = 3.812<br>c = 12.385	$Ca_{1.50}Sr_{0.50}CuO_3$	a = 3.481<br>b = 16.164<br>c = 3.880	a = 3.481<br>b = 16.144<br>c = 3.873			
LCuCa (**3**) &<br>LNiSr (**7**)	$CaSrCuO_3$	a = 3.390<br>b = 3.850<br>c = 12.496	a = 3.386<br>b = 3.826<br>c = 12.440	NiO	a = 4.177	a = 4.181			
LCuSr (**4**) &<br>LNiCa (**6**)	$CaSrCuO_3$	a = 3.390<br>b = 3.850<br>c = 12.496	a = 3.390<br>b = 3.827<br>c = 12.443	NiO	a = 4.177	a = 4.181			

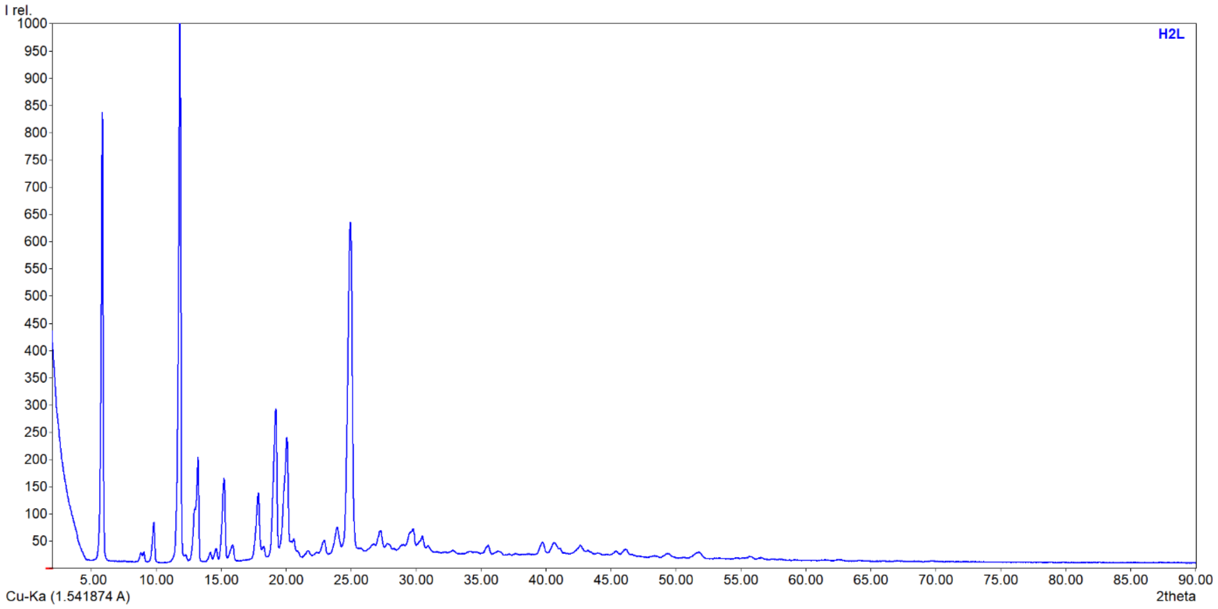

Figure S1. X-ray powder diffraction pattern of H_2L.

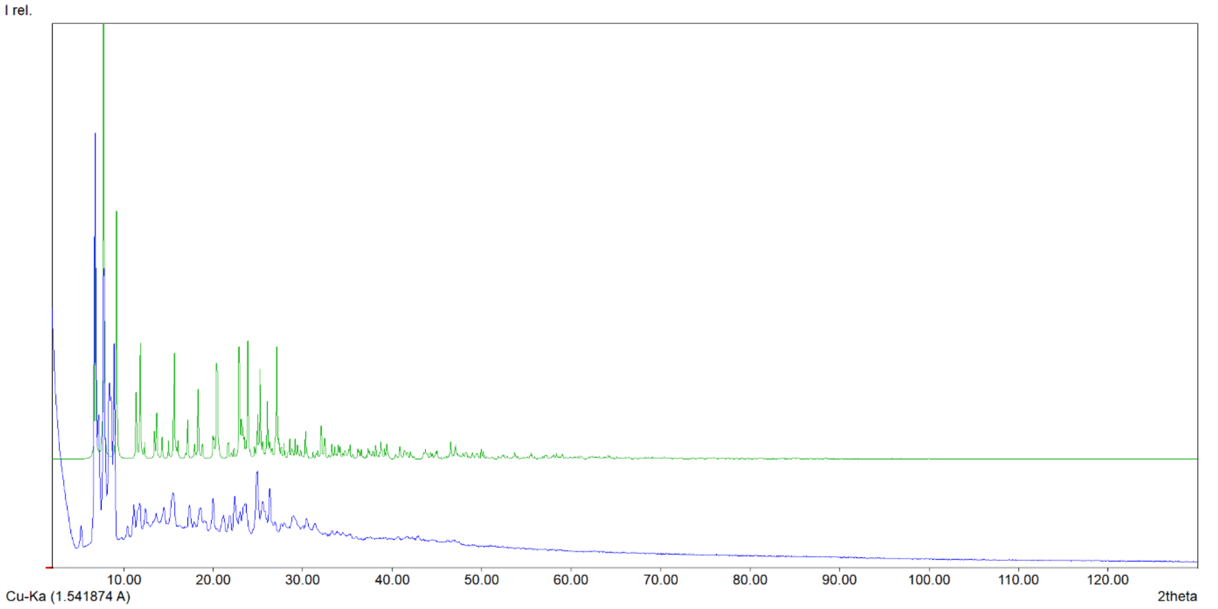

Figure S2. X-ray powder diffraction pattern of LCu (**1**) (blue) and the theoretical pattern (green).

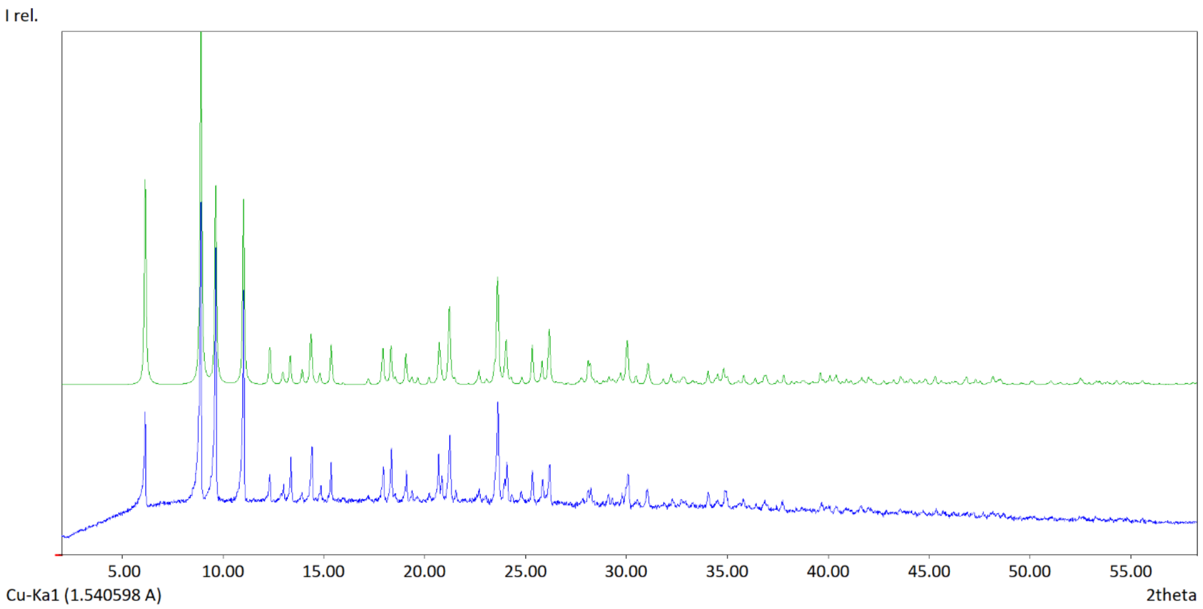

Figure S3. X-ray powder diffraction pattern of LCuCa (**3**) (blue) and the theoretical pattern (green).

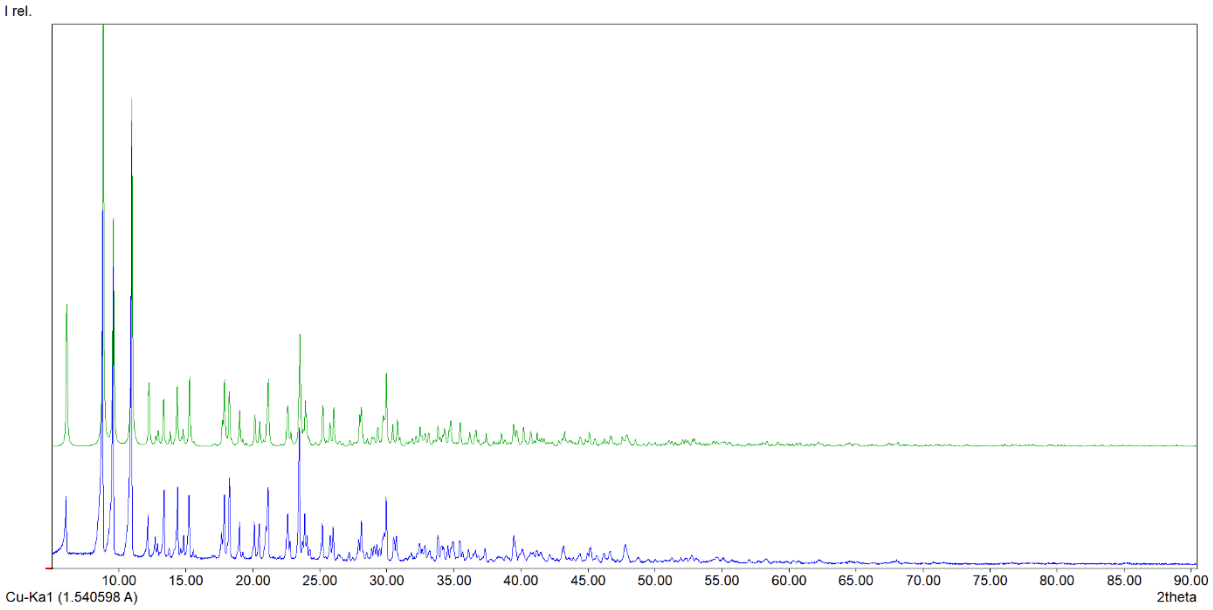

Figure S4. X-ray powder diffraction pattern of LCuSr (**4**) (blue) and the theoretical pattern (green).

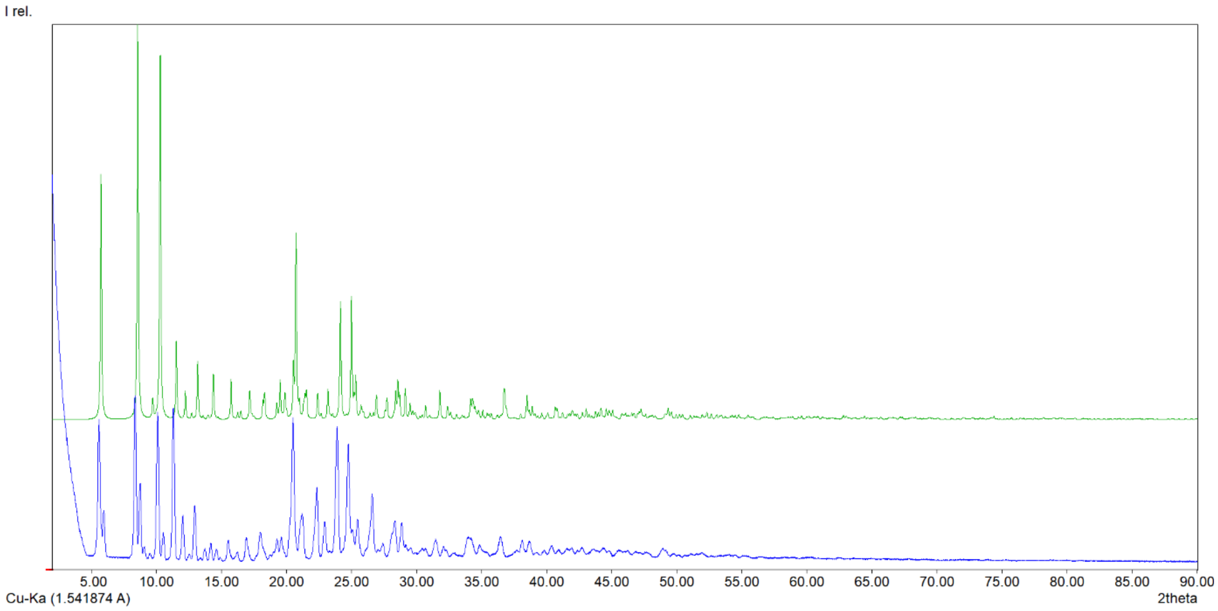

Figure S5. X-ray powder diffraction pattern of LCuBa (**5**) (blue) and the theoretical pattern (green).

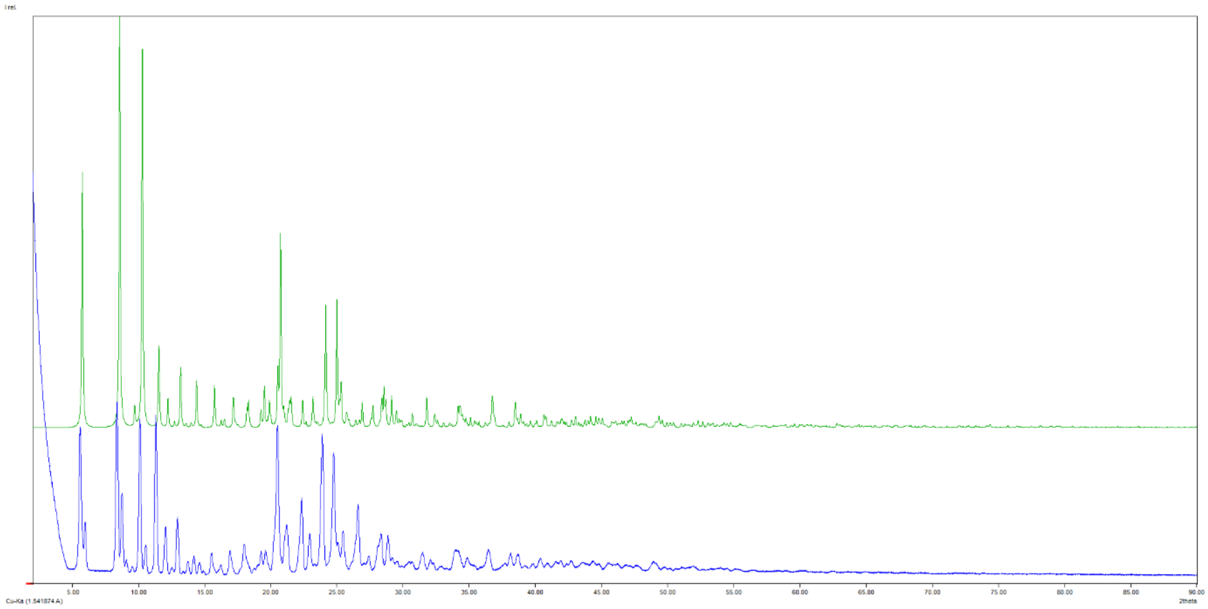

Figure S6. X-ray powder diffraction pattern of LNiCa (**6**) (blue) and the theoretical pattern (green).

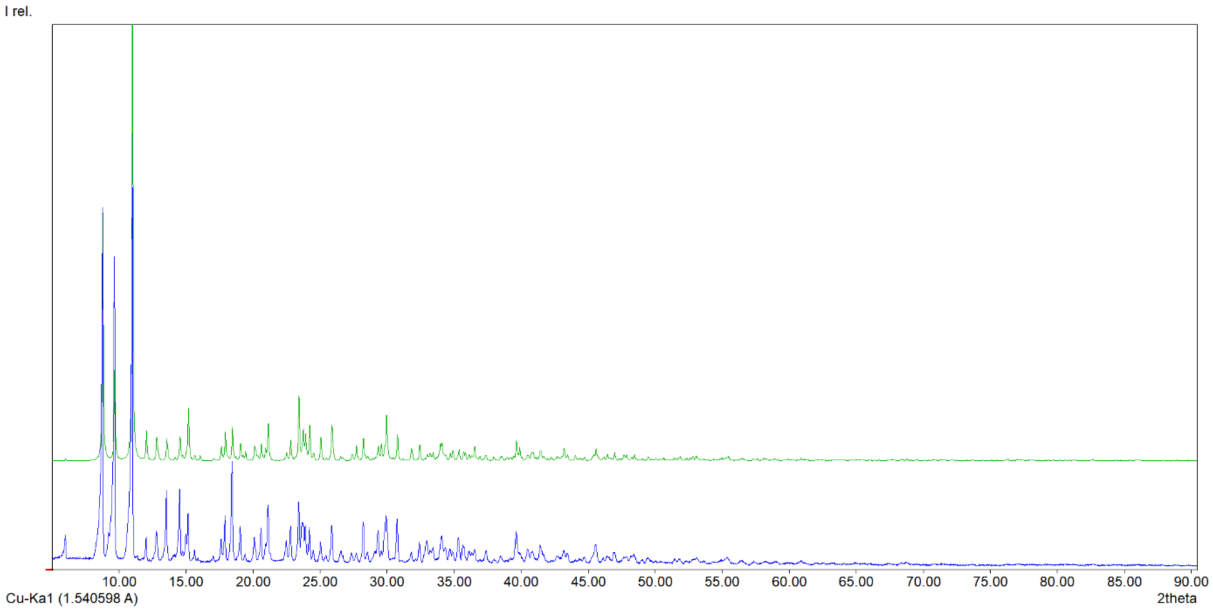

Figure S7. X-ray powder diffraction pattern of LNiSr (**7**) (blue) and the theoretical pattern (green).

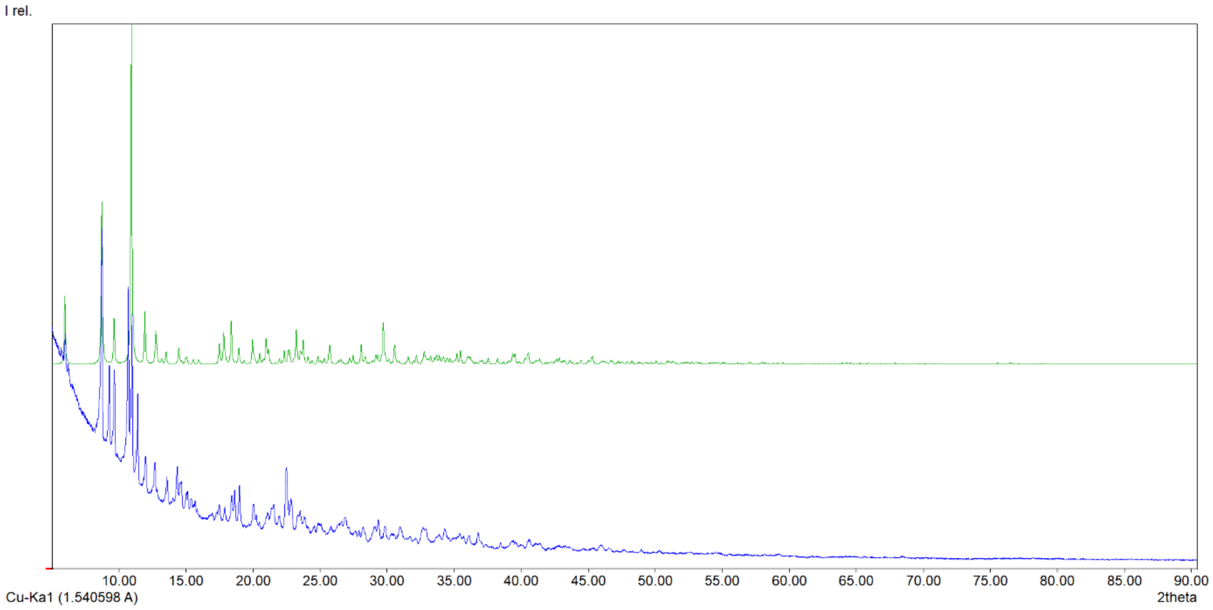

Figure S8. X-ray powder diffraction pattern of LNiBa (**8**) (blue) and the theoretical pattern (green).

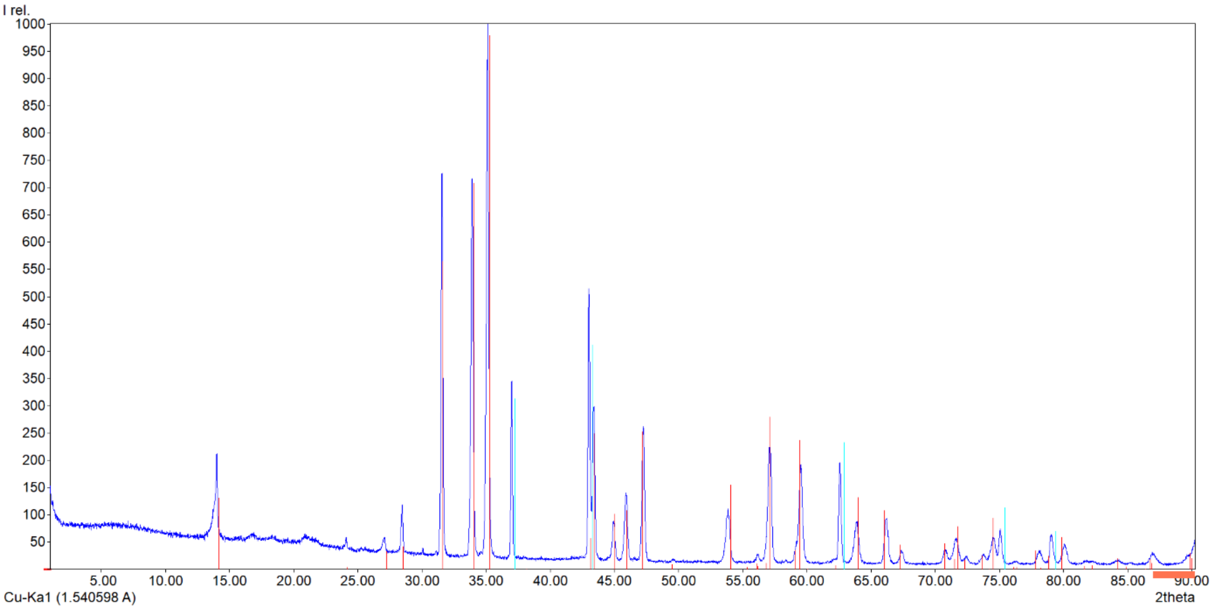

Figure S9. X-ray powder diffraction pattern of LCuCa (**3**) (blue) after calcination at 1000°C and the corresponding oxides (Ca_2CuO_3 (red), CuO (orange), and CaO (green)).

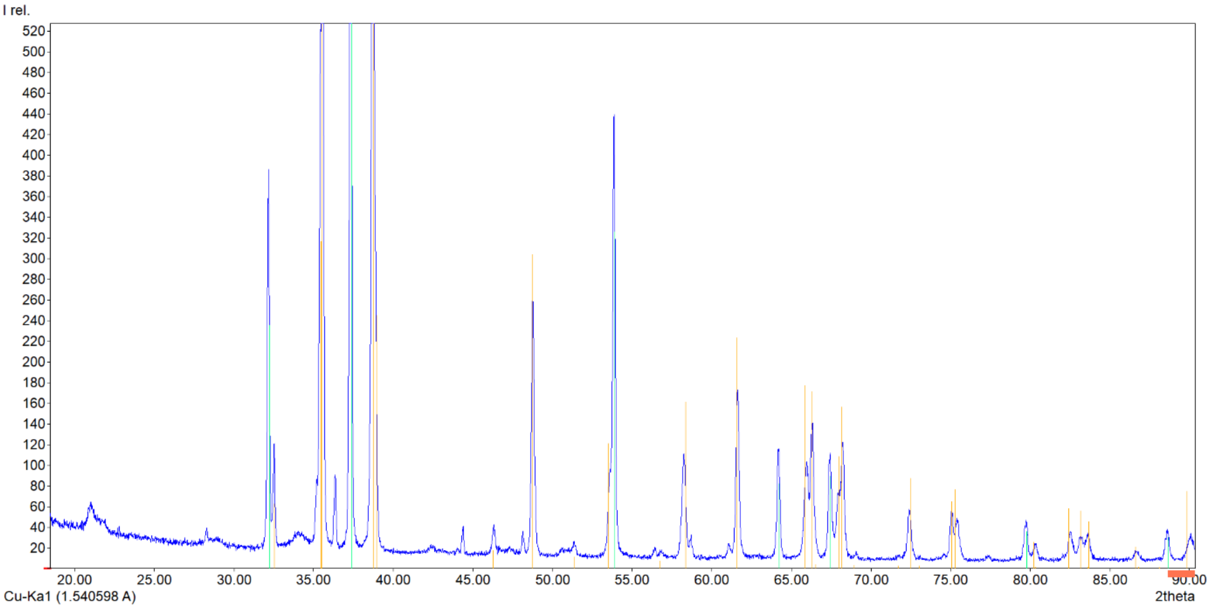

Figure S10. X-ray powder diffraction pattern of LCuCa (**3**) (blue) after calcination at 800°C and the corresponding oxides (CuO (orange) and CaO (green)).

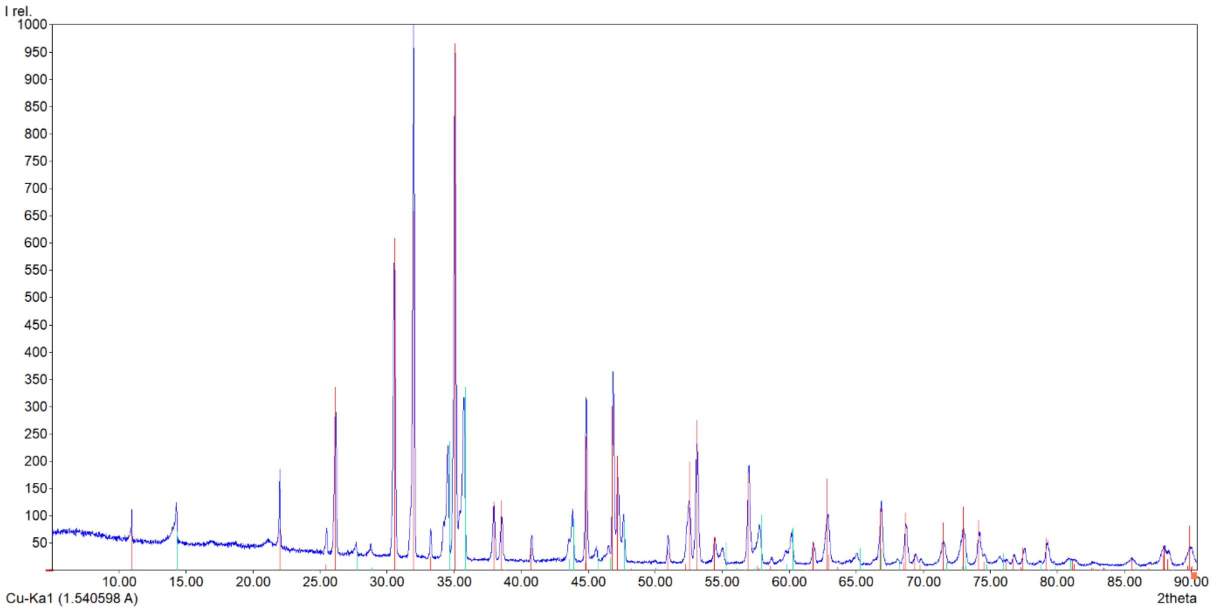

Figure S11. X-ray powder diffraction pattern of LCuSr (**4**) (blue) after calcination at 1000°C and the corresponding oxides ($SrCuO_2$ (green), Sr_2CuO_3 (red)).

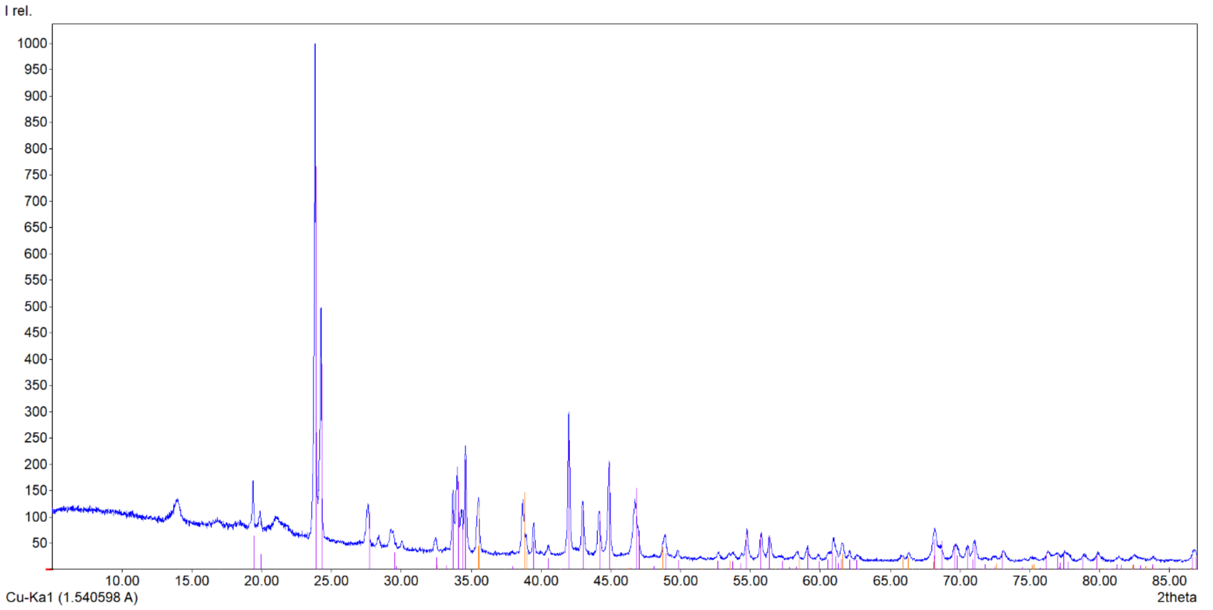

Figure S12. X-ray powder diffraction pattern of LCuBa (**5**) (blue) after calcination at 1000°C and the corresponding oxides ($BaCO_3$ (purple), CuO (orange)).

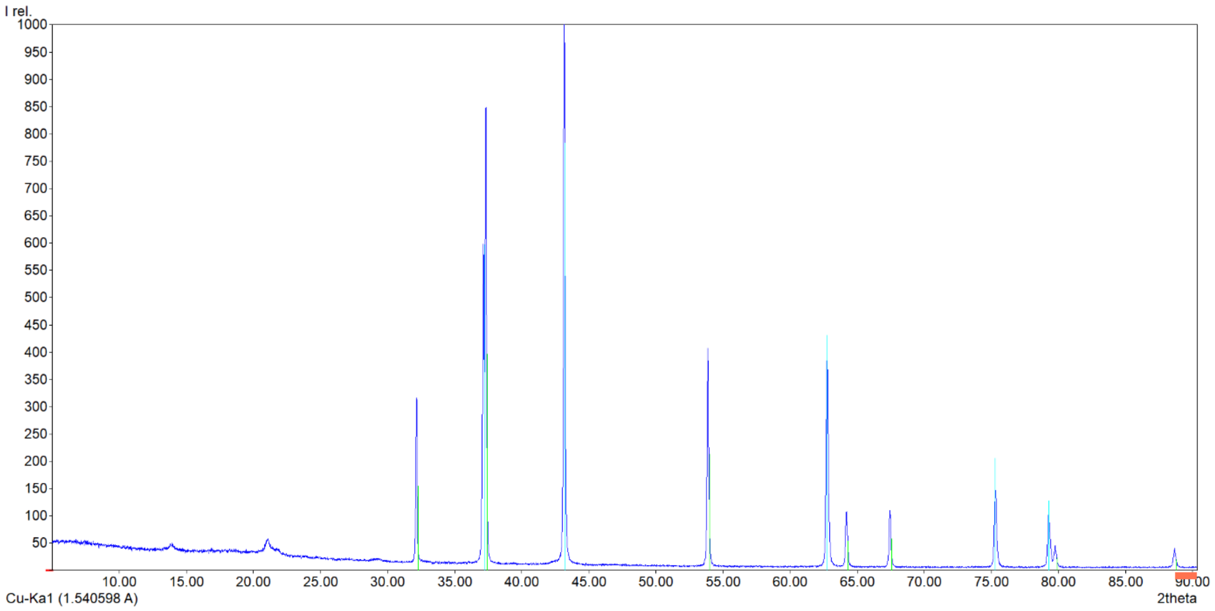

Figure S13. X-ray powder diffraction pattern of LNiCa (**6**) (blue) after calcination at 1000°C and the corresponding oxides (CaO (green), NiO (light blue)).

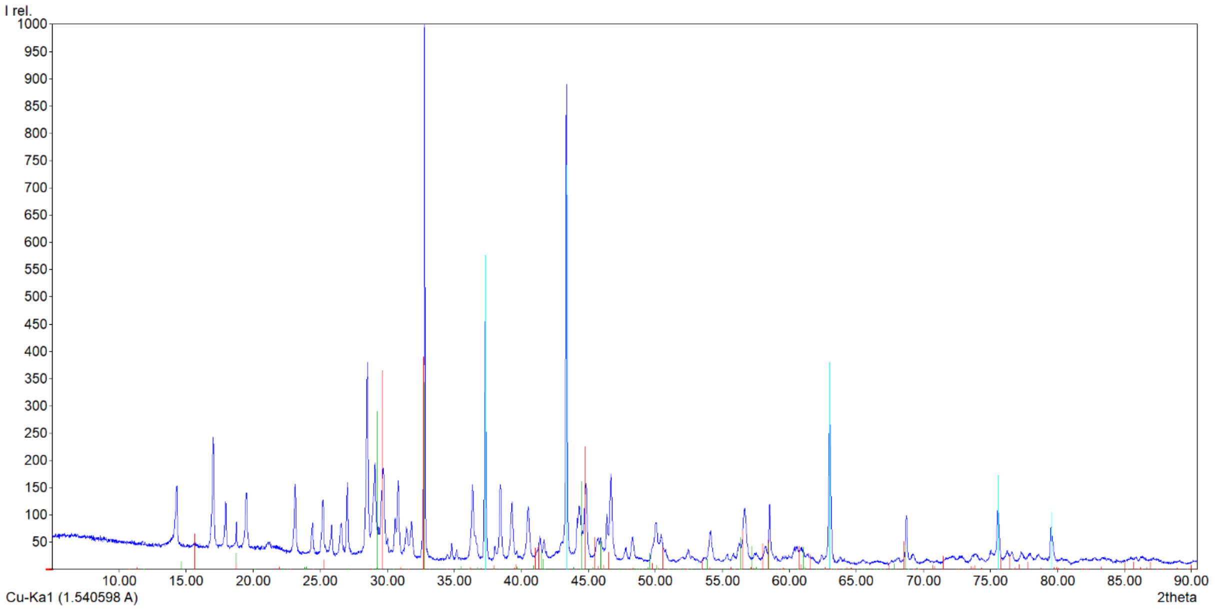

Figure S14. X-ray powder diffraction pattern of LNiSr (**7**) (blue) after calcination at 1000°C and the corresponding oxides ($Ni_{6.64}Sr_9O_{21}$ (green), $Ni_{2.5}Sr_4O_9$ (red), NiO (light blue)).

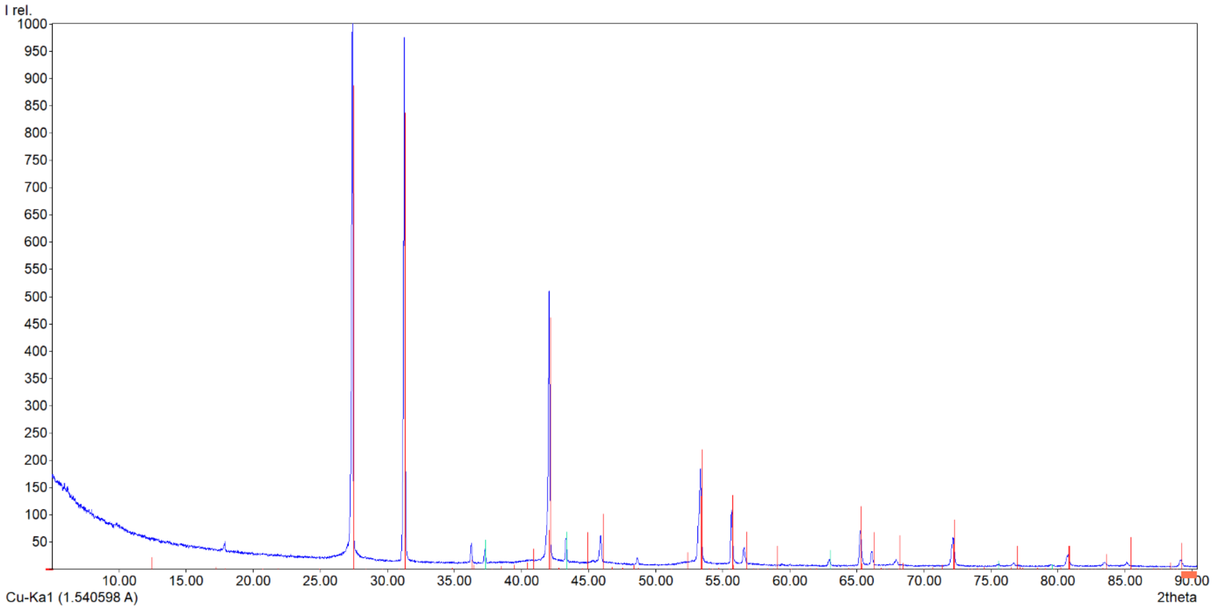

Fi gure S15. X-ray powder diffraction pattern of LNiBa (**8**) (blue) after calcination at 1000°C and the corresponding oxides ($Ni_5Ba_6O_{15}$ (red), NiO (light blue)).

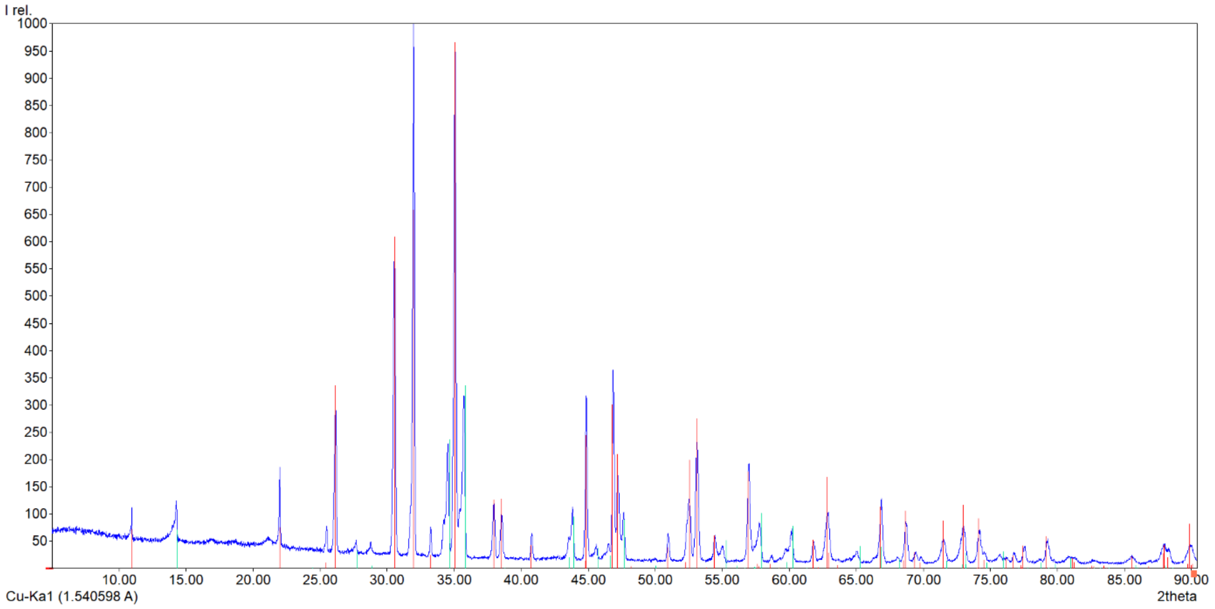

Figure S16. X-ray powder diffraction pattern of the LCuCa (**3**)/LCuSr (**4**) mixture (blue) after calcination at 1000°C and the corresponding oxides ($Ca_{0.38}Sr_{0.62}CuO_2$ (red), $Ca_{1.50}Sr_{0.50}CuO_3$ (green)).

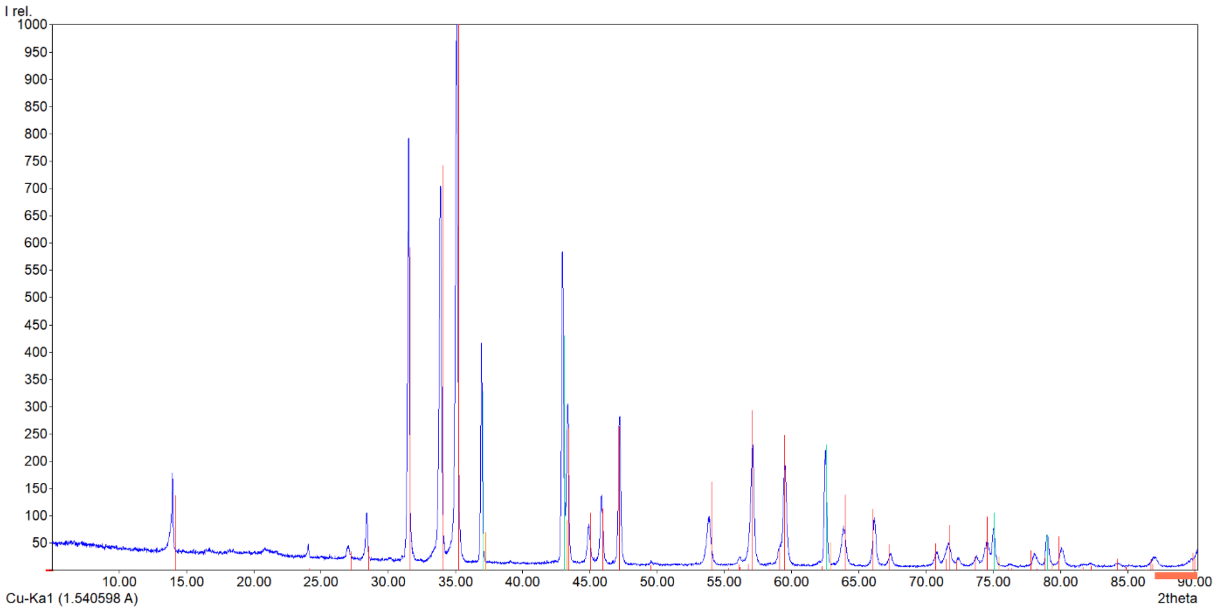

Figure S17. X-ray powder diffraction pattern of the LCuCa (**3**)/LNiSr (**7**) mixture (blue) after calcination at 1000°C and the corresponding oxides ($CaSrCuO_3$ (red), NiO (light blue)).

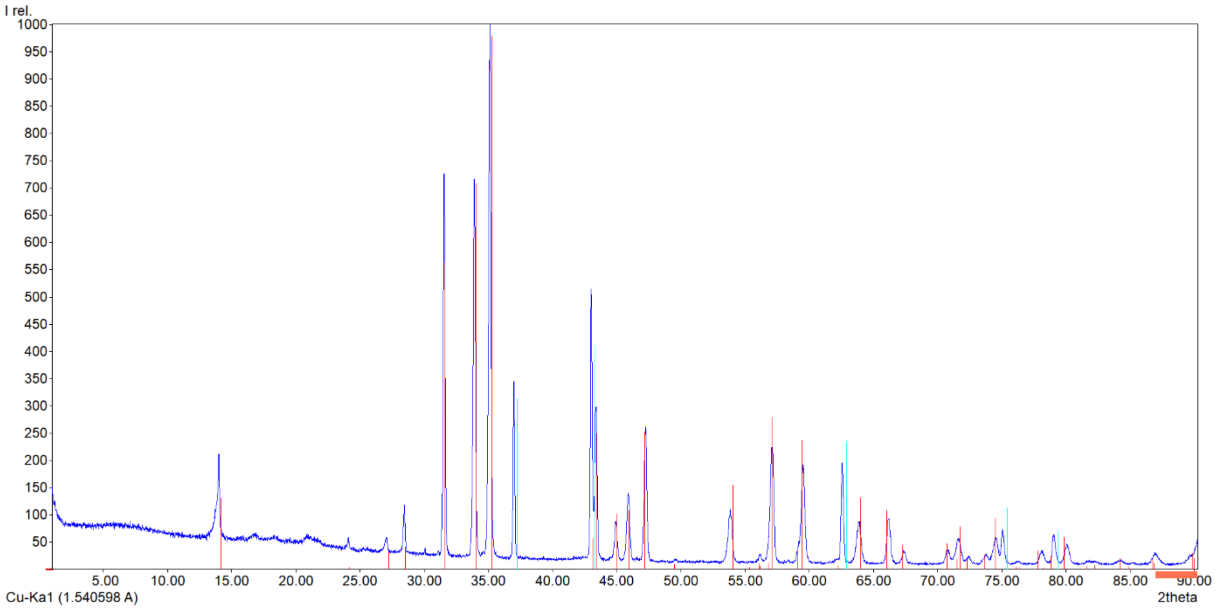

Figure S18. X-ray powder diffraction pattern of the LCuSr (**4**)/LNiCa (**6**) mixture (blue) after calcination at 1000°C and the corresponding oxides ($CaSrCuO_3$ (red), NiO (light blue)).

Thermogravimetric analysis (TGA)

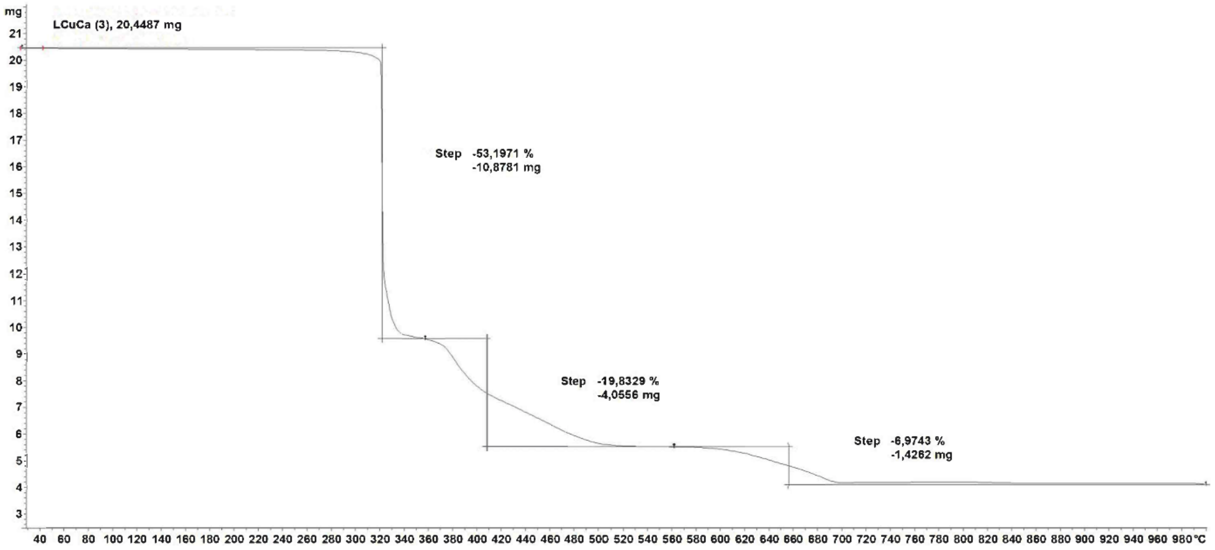

Figure S19. TGA spectrum of LCuCa (**3**).

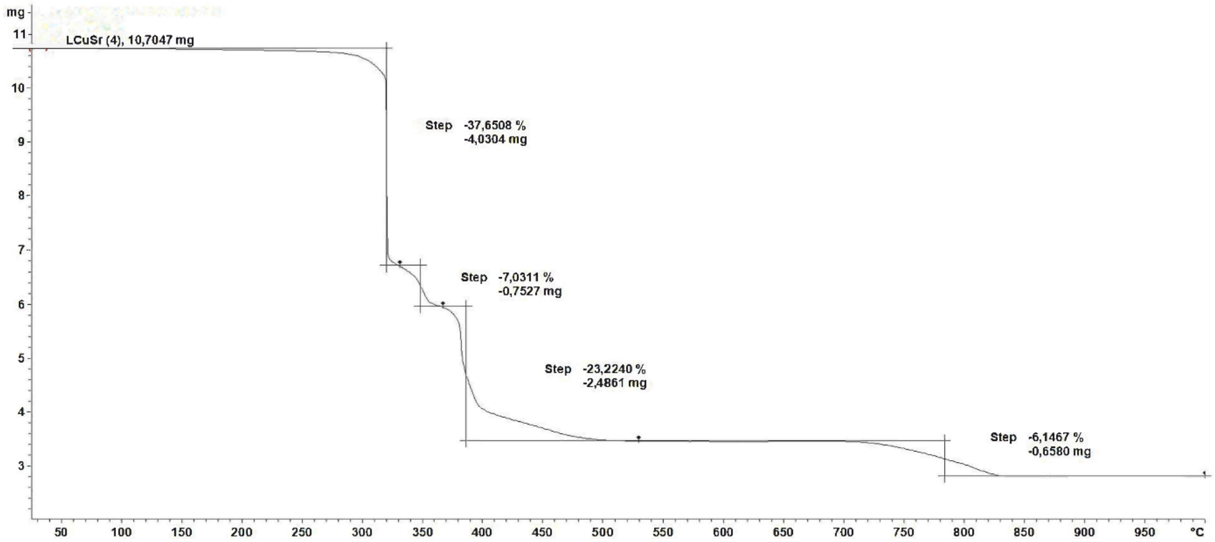

Figure S20. TGA spectrum of LCuSr (**4**).

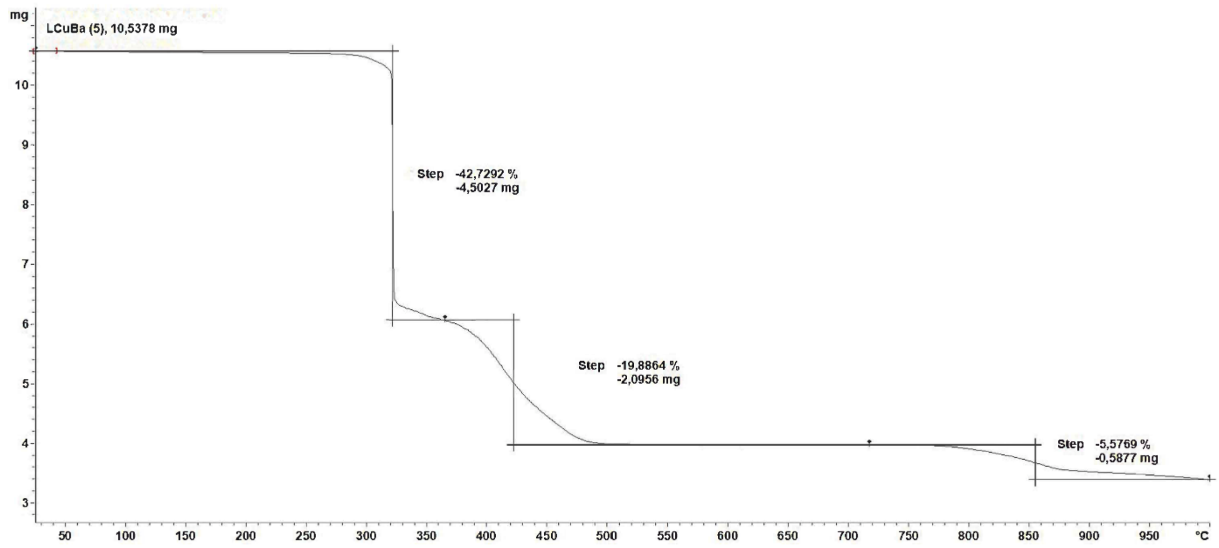

Figure S21. TGA spectrum of LCuBa (**5**).

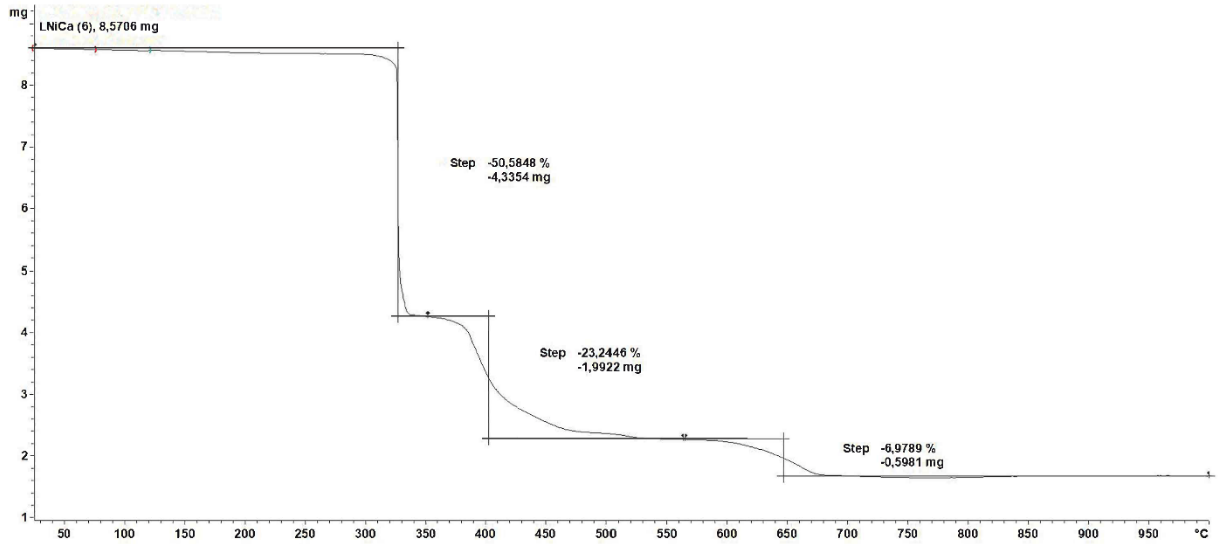

Figure S22. TGA spectrum of LNiCa (**6**).

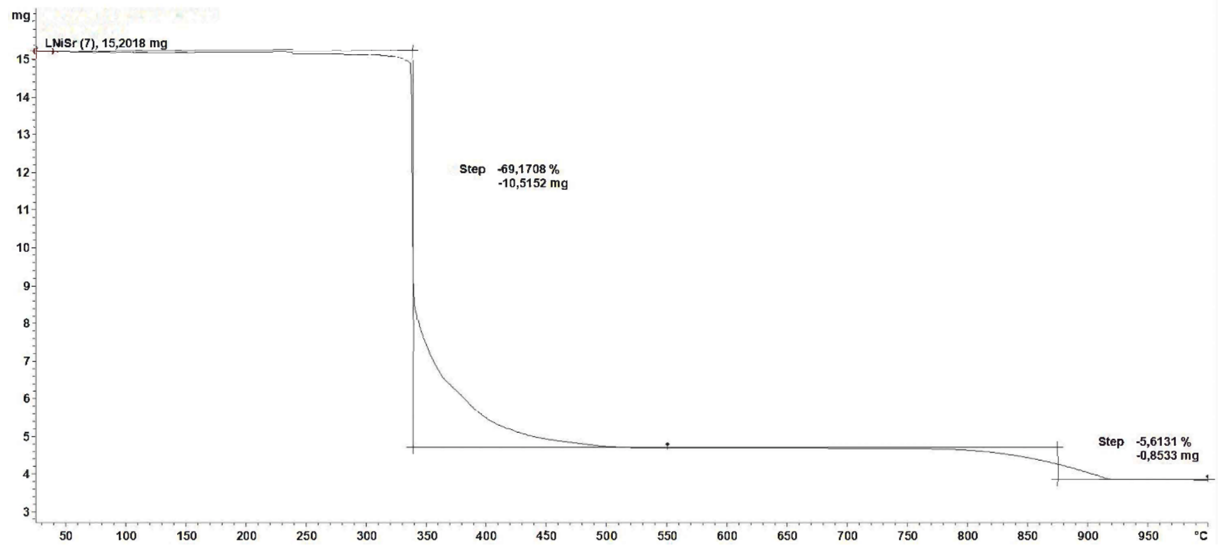

Figure S23. TGA spectrum of LNiSr (**7**).

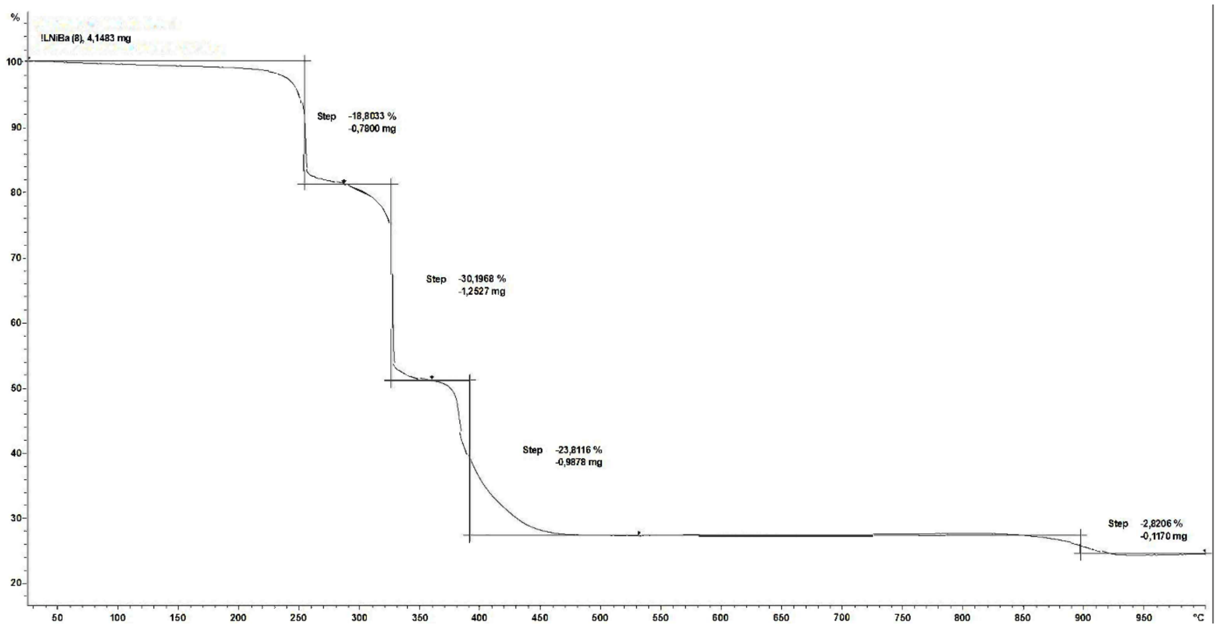

Figure S24. TGA spectrum of LNiBa (**8**).

SEM Images

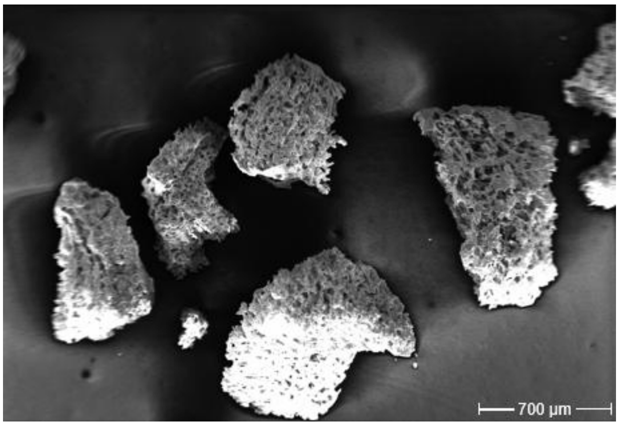

Figure S25. SEM image of calcined LCuCa (1000°C) without coating.

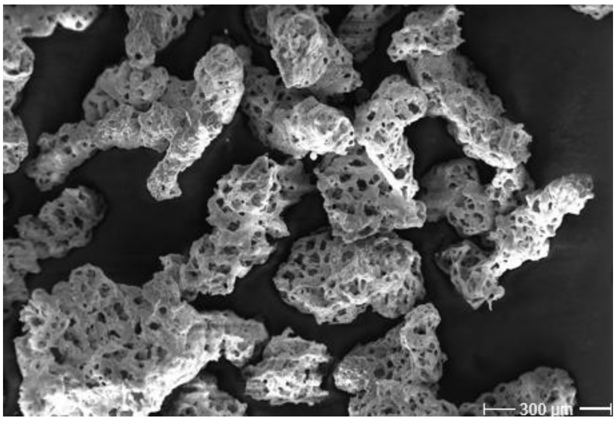

Figure S26. SEM image of calcined LCuSr (1000°C) without coating.

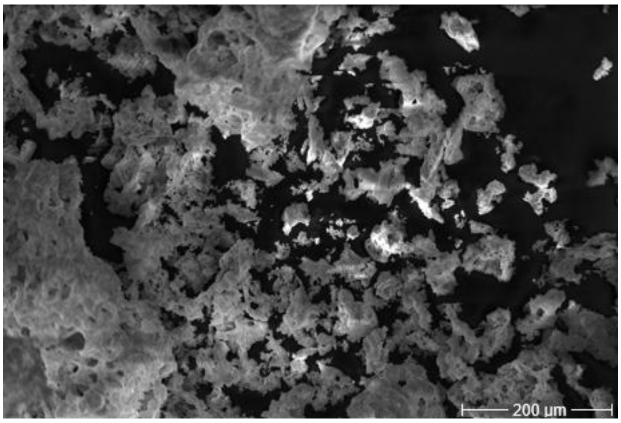

Figure S27. SEM image of calcined LNiSr (1000°C) without coating.

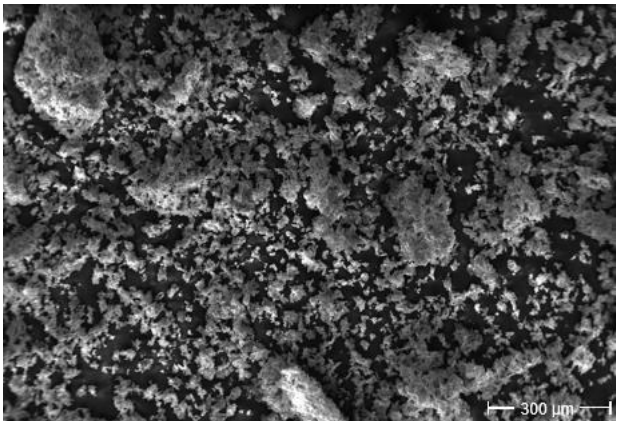

Figure S28. SEM image of calcined LNiBa (1000°C) without coating.

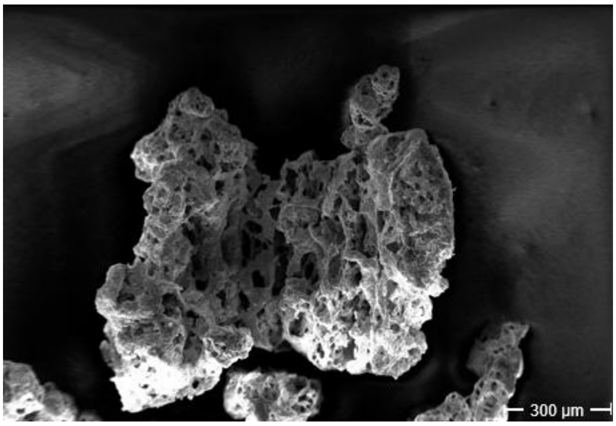

Figure S29. SEM image of calcined mixture of LCuCa/LCuSr (1000°C) without coating.

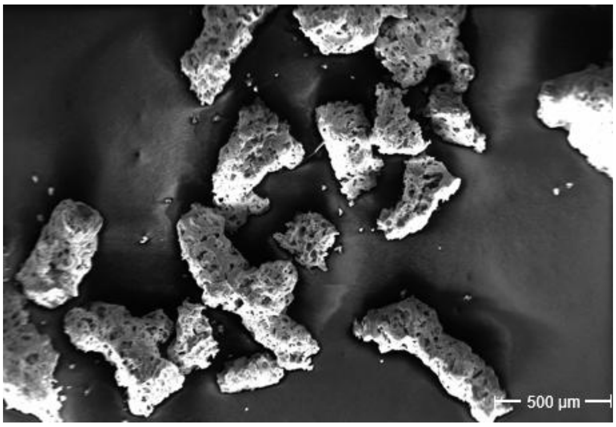

Figure S30. SEM image of calcined mixture of LCuCa/LNiSr (1000°C) without coating.

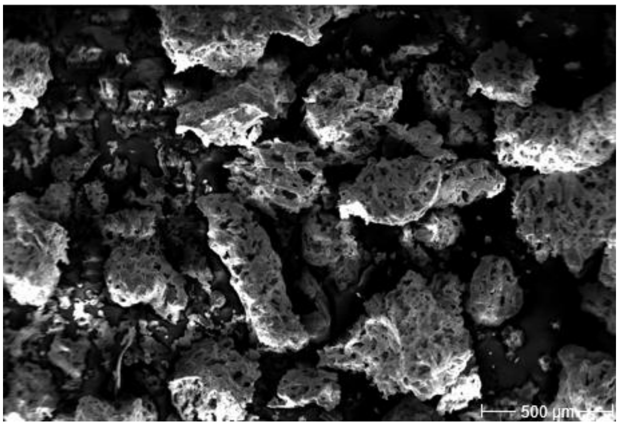

Figure S31. SEM image of calcined mixture of LCuSr/LNiCa (1000°C) without coating.

^{1}H and ^{13}C NMR Spectra

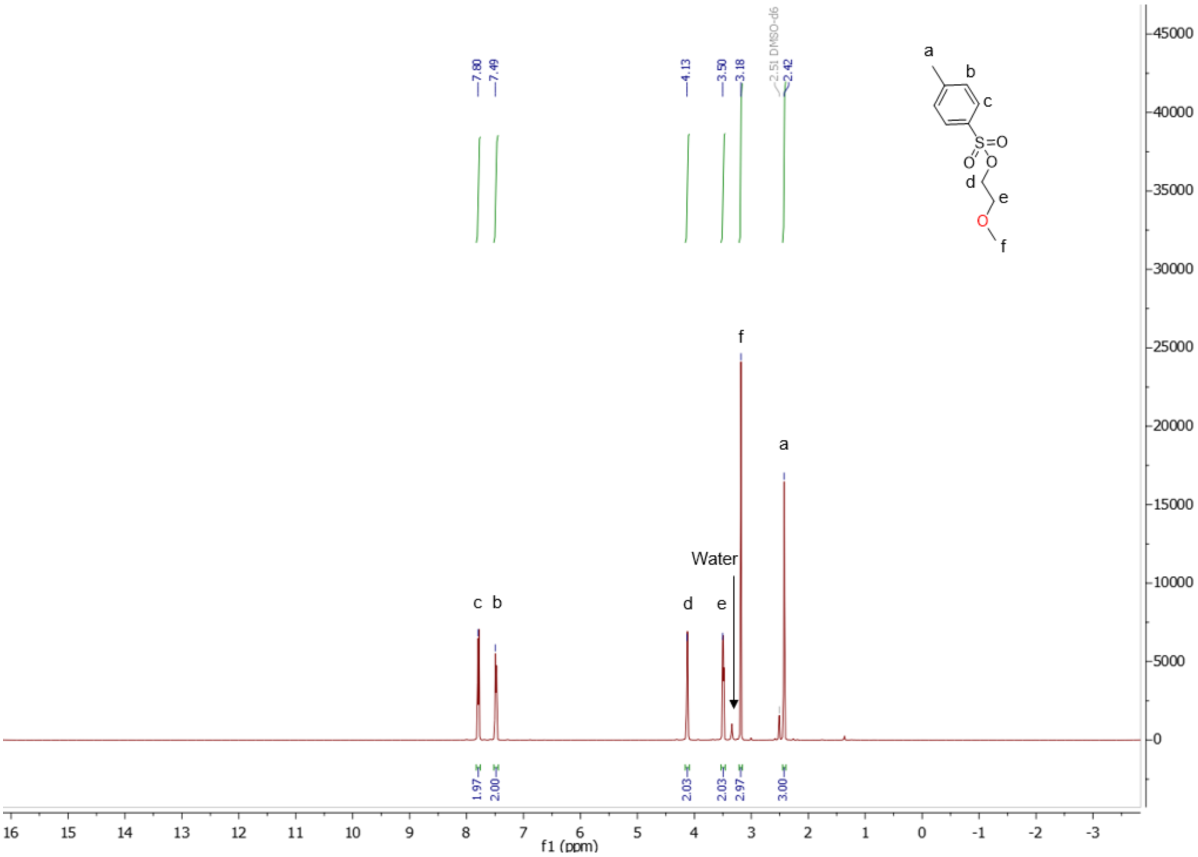

Figure S32. ^{1}H NMR spectrum of L' in DMSO-*d*6.

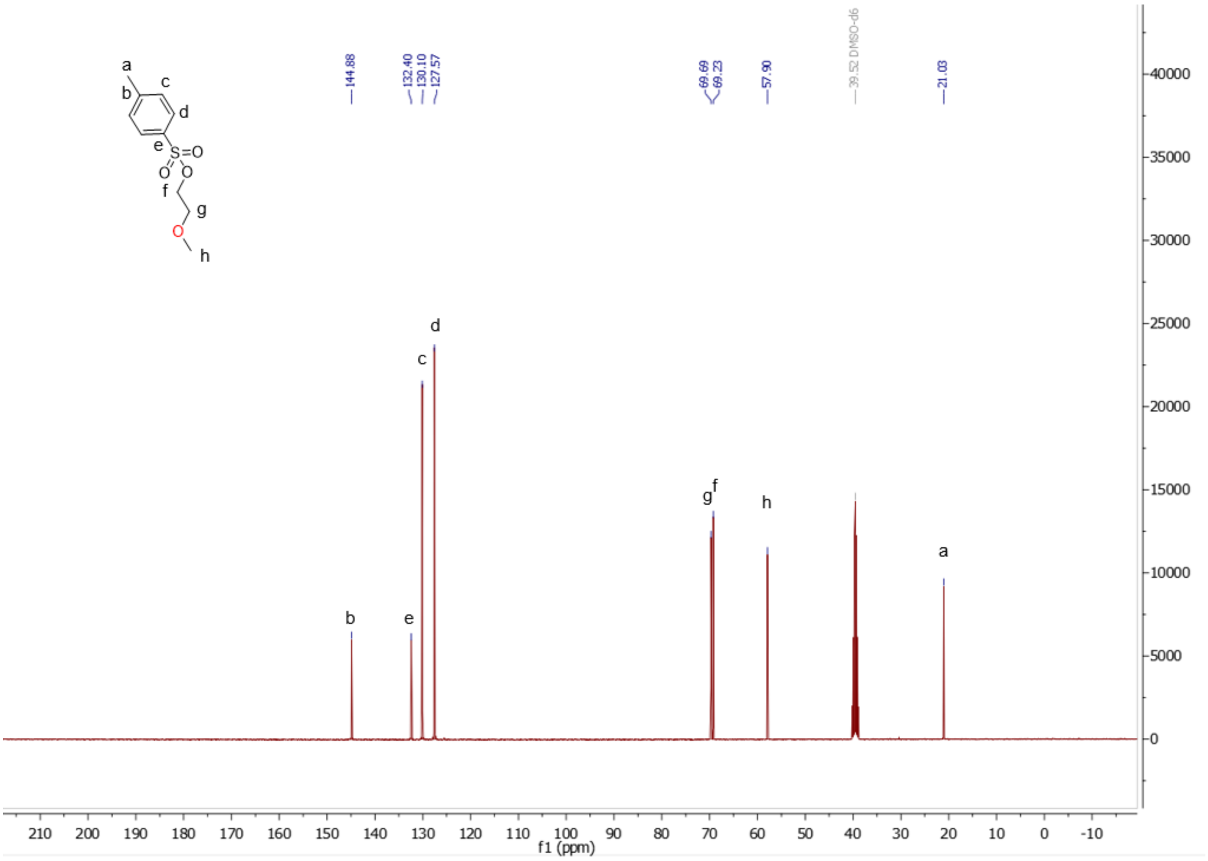

Figure S33. ^{13}C NMR spectrum of L' in DMSO-*d*6.

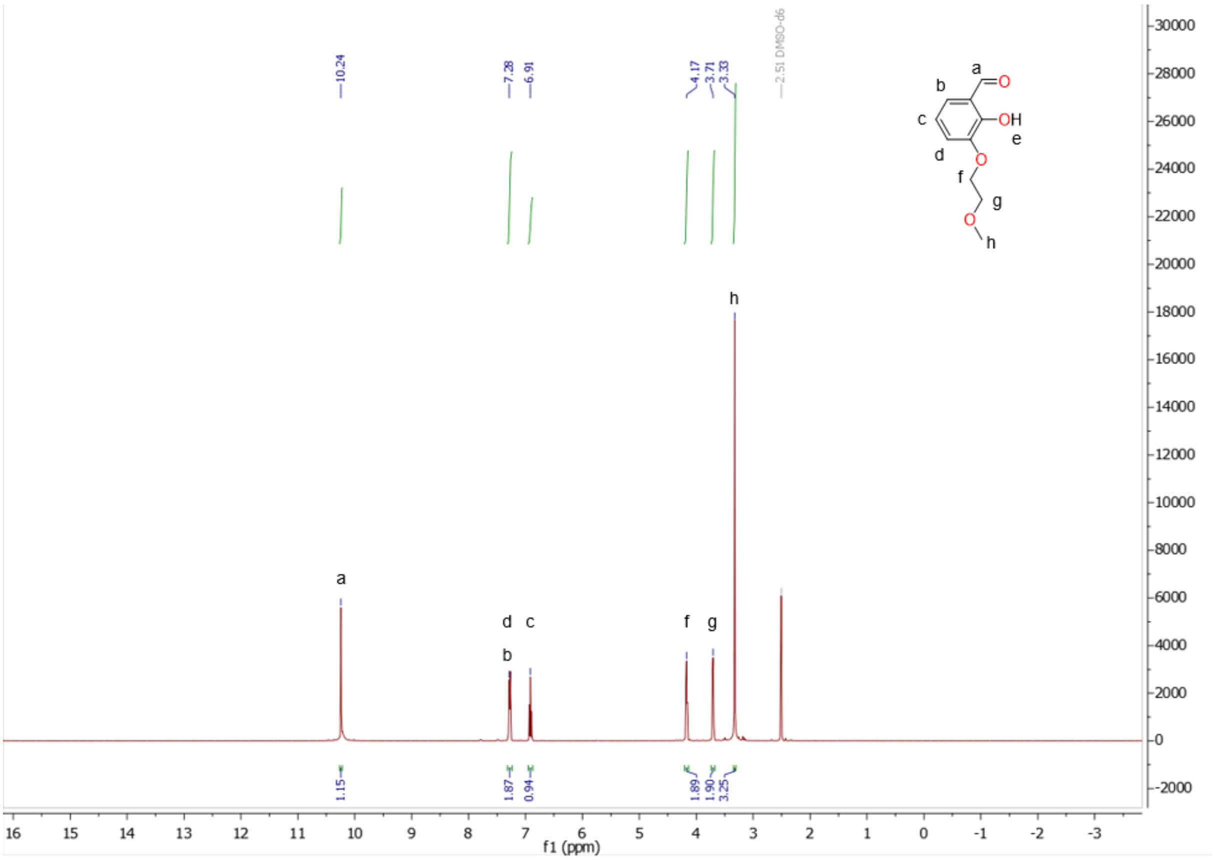

Figure S34. ^{1}H NMR spectrum of L'' in DMSO-*d*6.

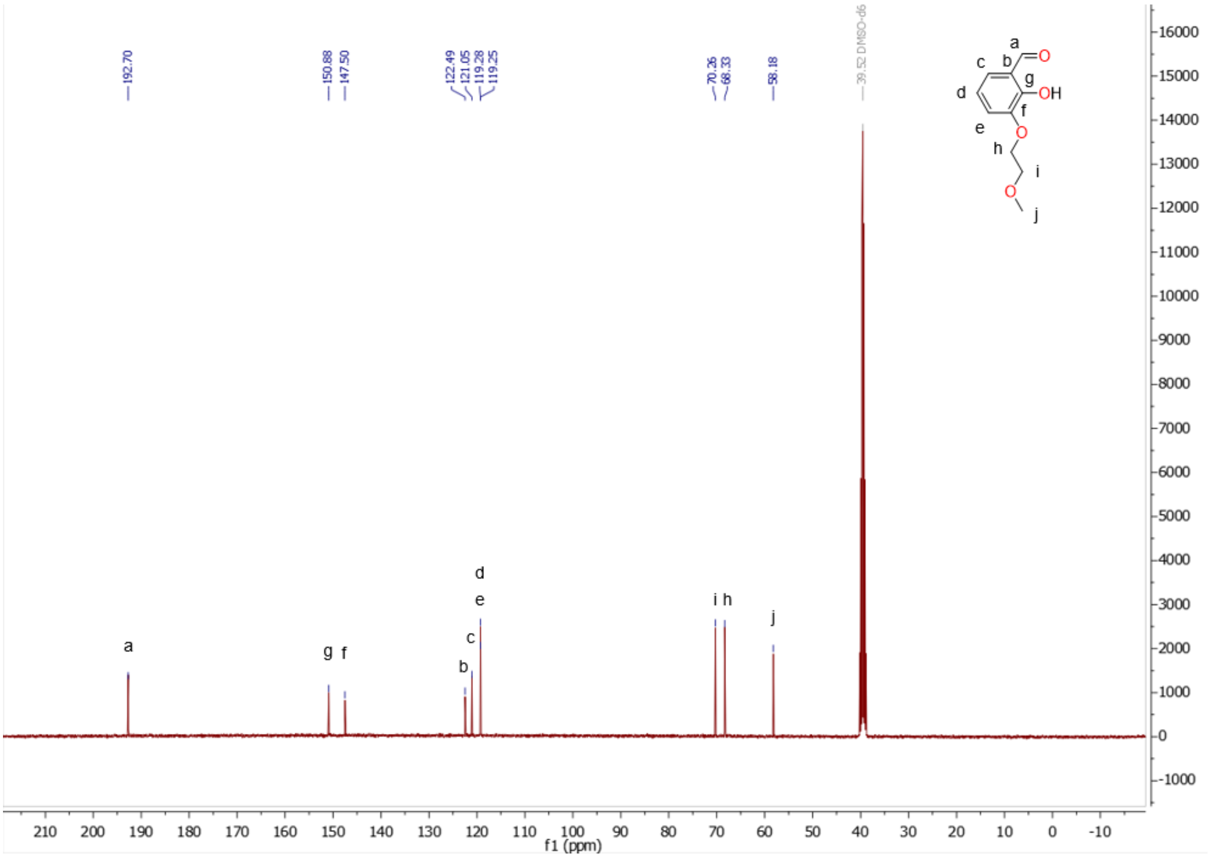

Figure S35. ^{13}C NMR spectrum of L'' in DMSO-*d*6.

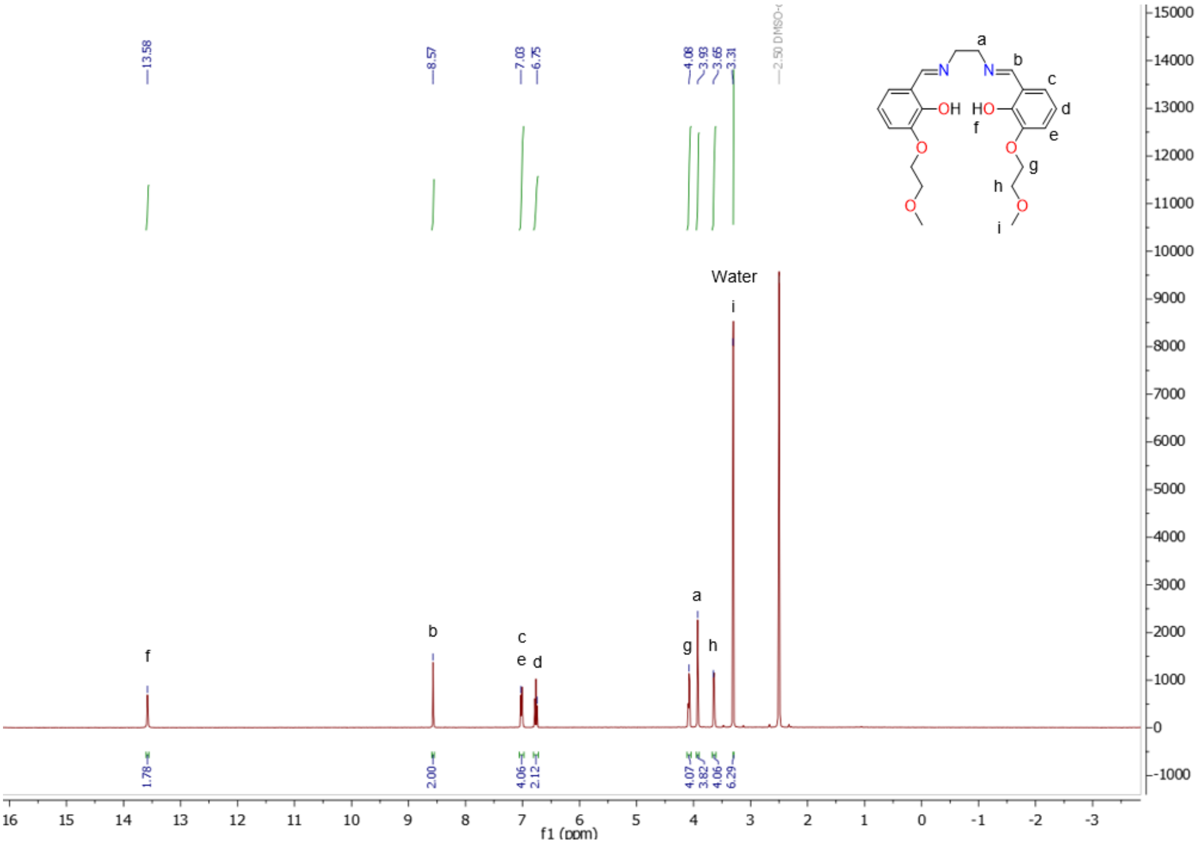

Figure S36. ^{1}H NMR spectrum of H_2L in DMSO-*d*6.

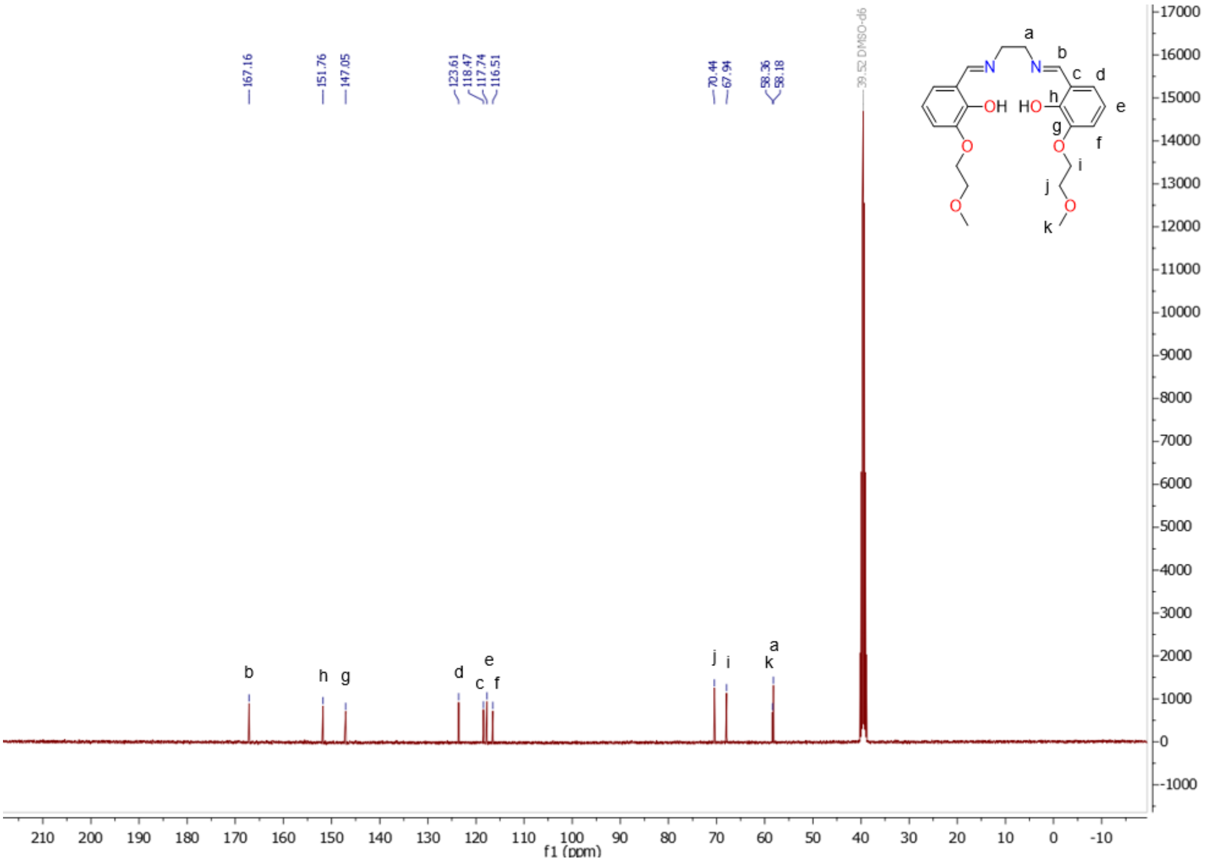

Figure S37. ^{13}C NMR spectrum of H_2L in DMSO-*d*6.

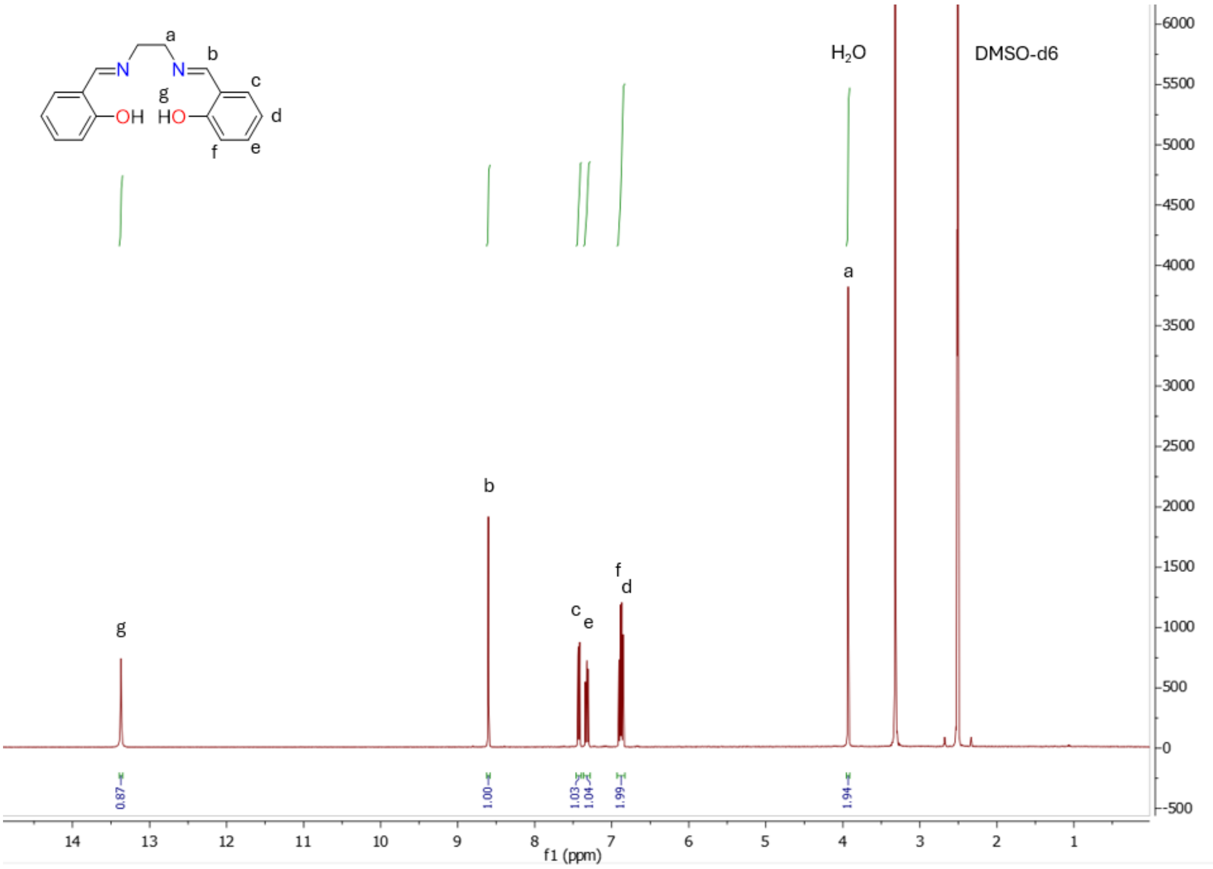

Figure S38. ^{1}H NMR spectrum of the original salen ligand in DMSO-*d*6.

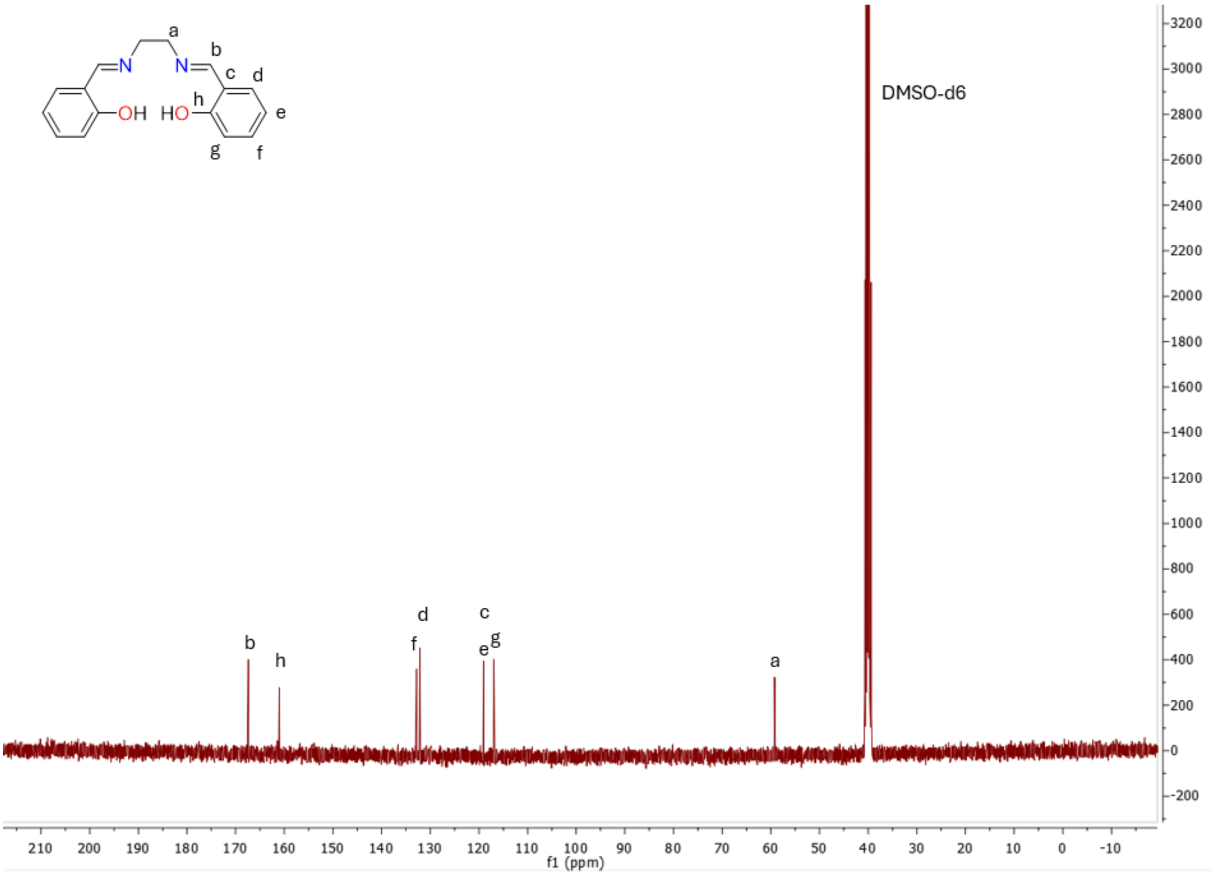

Figure S39. ^{13}C NMR spectrum of the original salen ligand in DMSO-*d*6.

ESI-MS Spectra

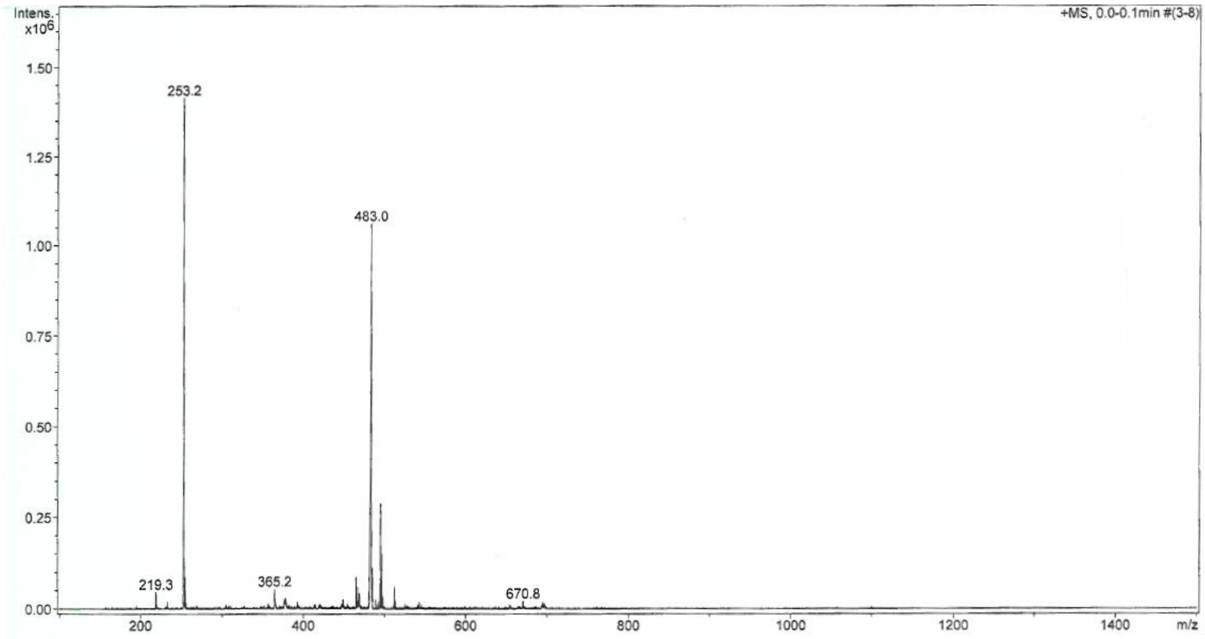

Figure S40. ESI-MS spectra of the precursor L’.

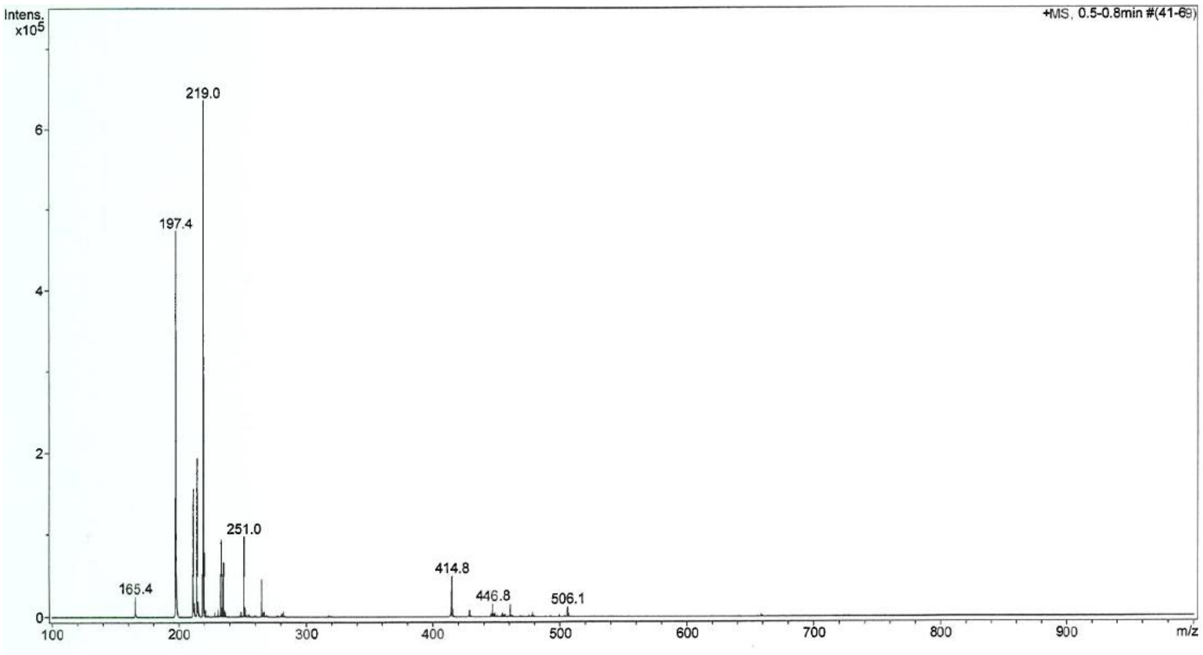

Figure S41. ESI-MS spectra of the precursor L''.

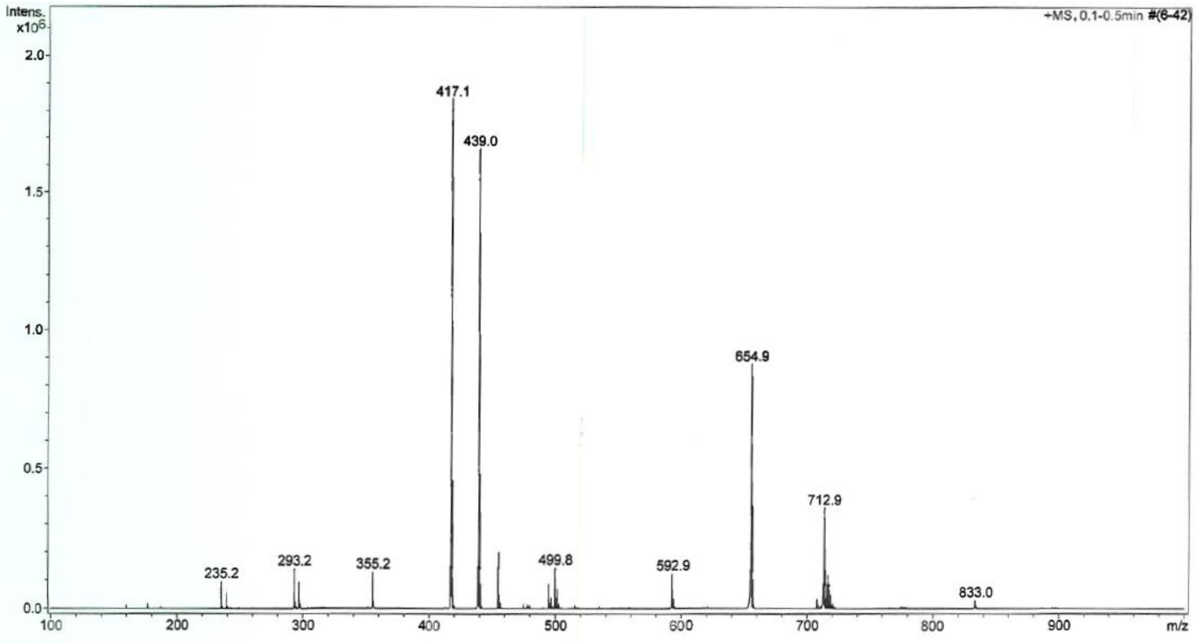

Figure S42. ESI-MS spectra of the ligand H_2L.

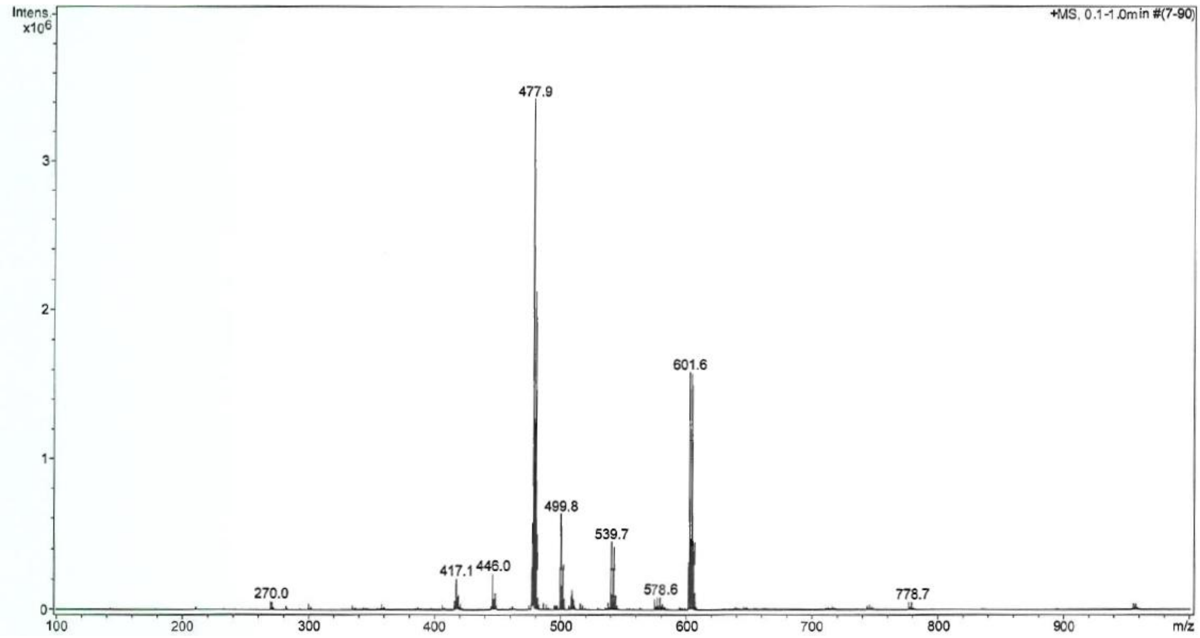

Figure S43. ESI-MS spectra of the LCu complex (**1**).

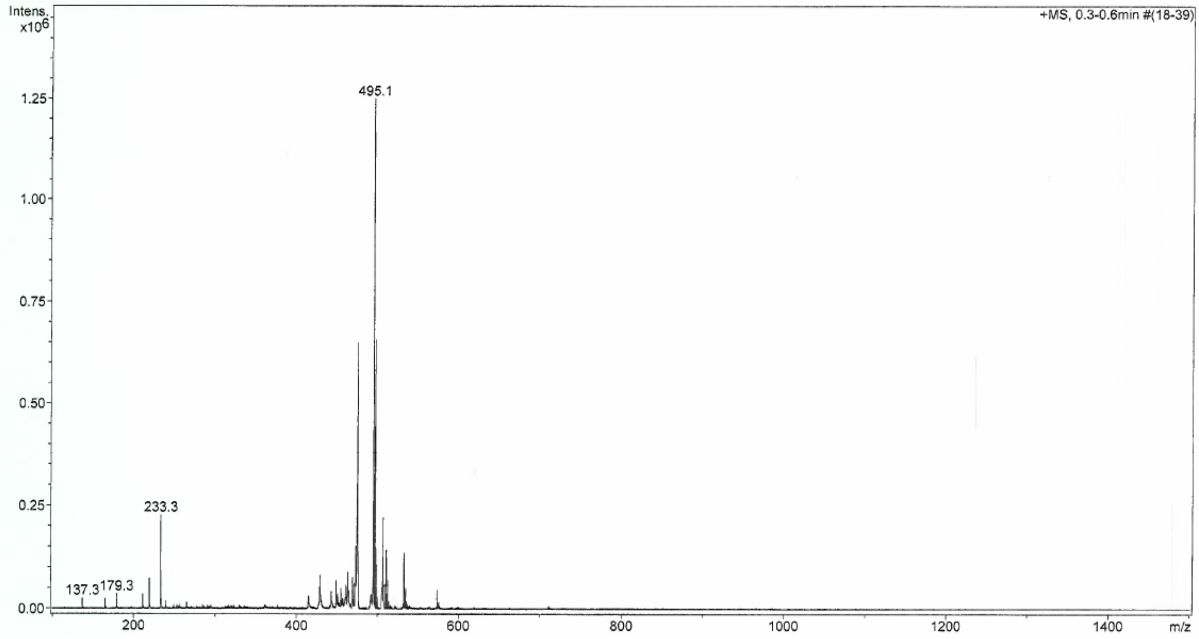

Figure S44. ESI-MS spectra of the LNi complex (**2**).

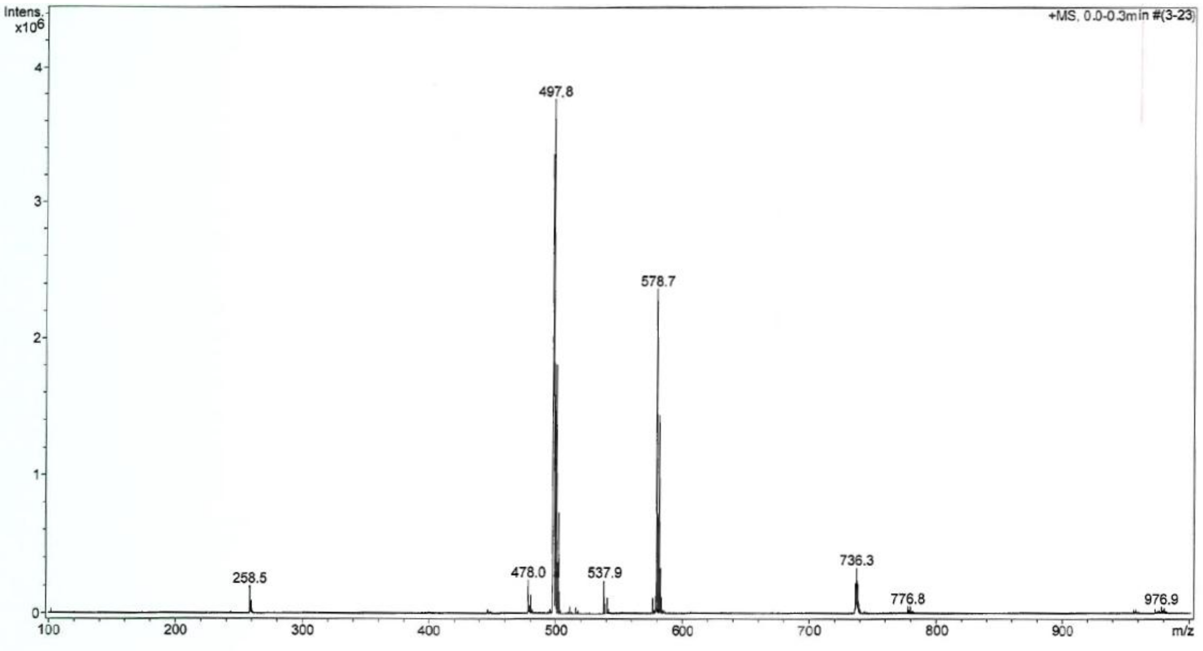

Figure S45. ESI-MS spectra of the LCuCa complex (**3**).

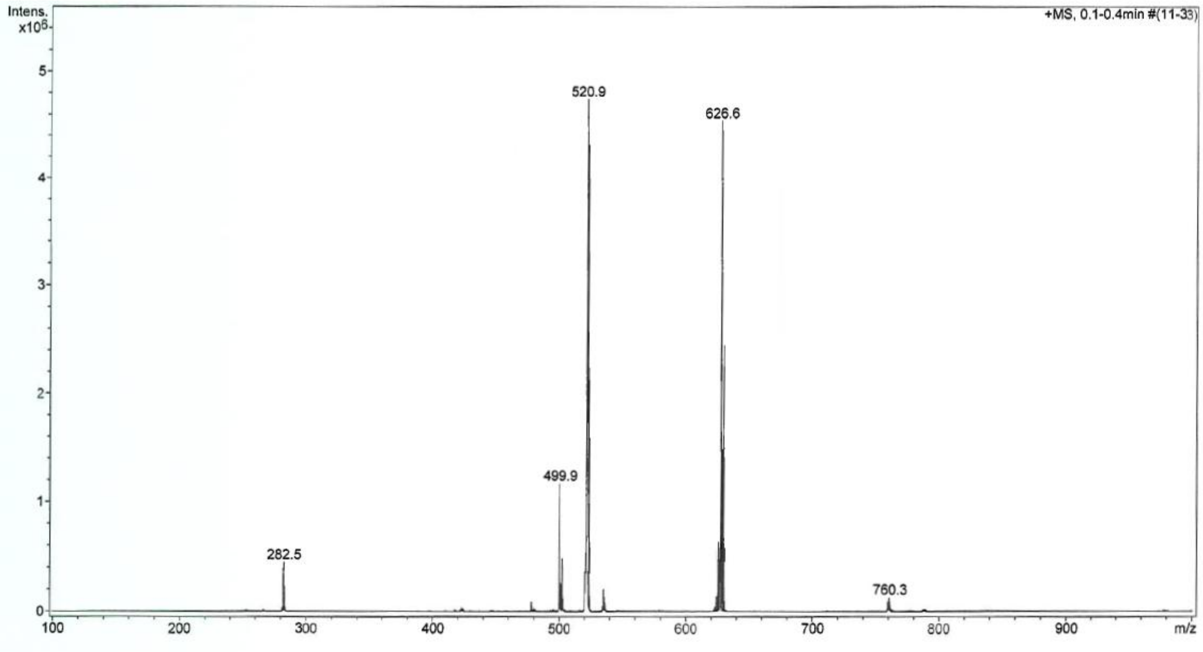

Figure S46. ESI-MS spectra of the LCuSr complex (**4**).

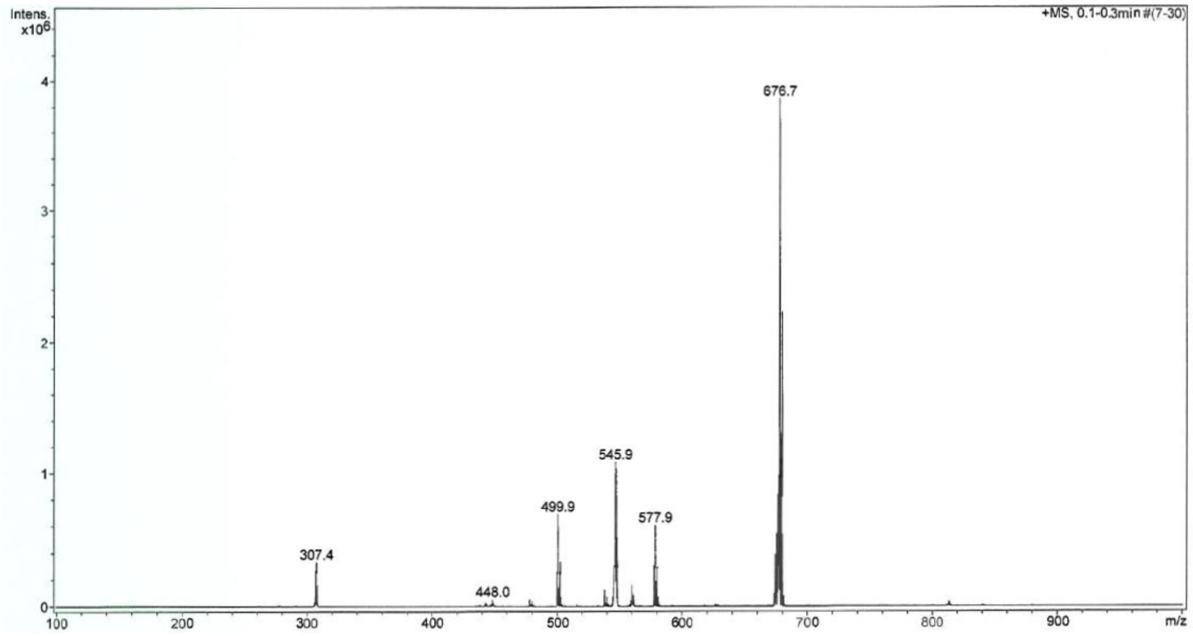

Figure S47. ESI-MS spectra of the LCuBa complex (**5**).

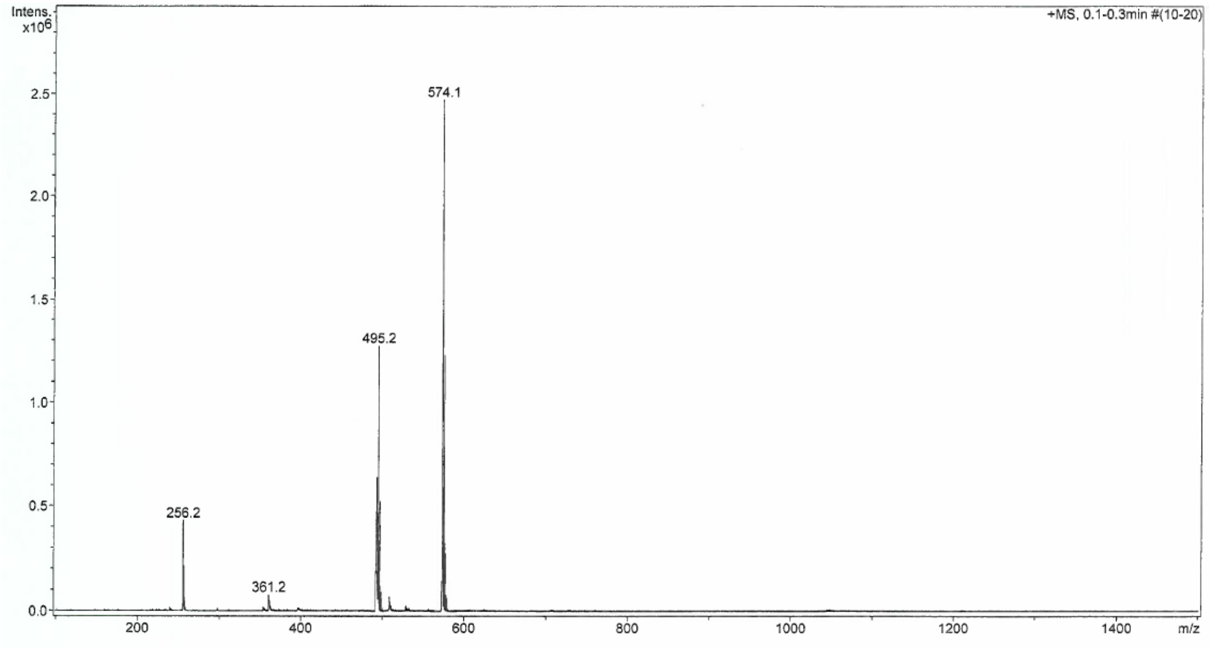

Figure S48. ESI-MS spectra of the LNiCa complex (**6**).

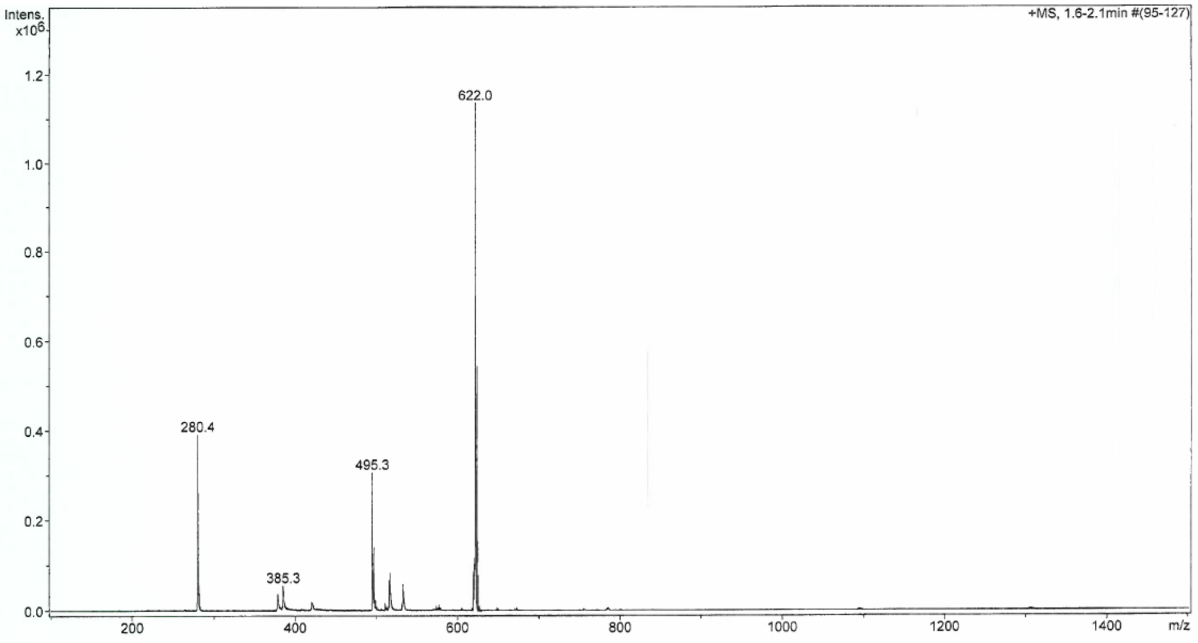

Figure S49. ESI-MS spectra of the LNiSr complex (**7**).

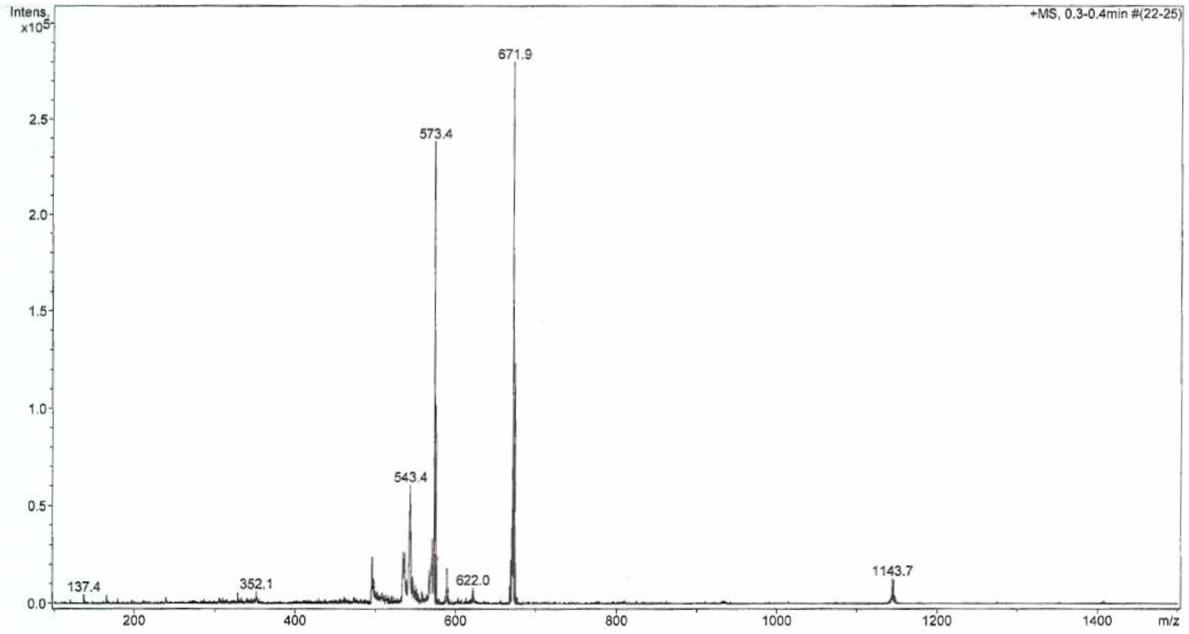

Figure S50. ESI-MS spectra of the LNiBa complex (**8**).
